# The contemporary nexus of medicines security and bioprospecting: a future perspective for prioritizing the patient

**DOI:** 10.1007/s13659-024-00431-5

**Published:** 2024-01-25

**Authors:** Geoffrey A. Cordell

**Affiliations:** 1 Natural Products Inc., 1320 Ashland Avenue, Evanston, IL 60201 USA; 2 Department of Pharmaceutics, College of Pharmacy, University of Florida, Gainesville, FL 32610 USA

**Keywords:** Medicines security, Traditional medicine, Optimizing resources, Sustainability, Defossilization, Action initiatives

## Abstract

Reacting to the challenges presented by the evolving nexus of environmental change, defossilization, and diversified natural product bioprospecting is vitally important for advancing global healthcare and placing patient benefit as the most important consideration. This overview emphasizes the importance of natural and synthetic medicines security and proposes areas for global research action to enhance the quality, safety, and effectiveness of sustainable natural medicines. Following a discussion of some contemporary factors influencing natural products, a rethinking of the paradigms in natural products research is presented in the interwoven contexts of the Fourth and Fifth Industrial Revolutions and based on the optimization of the valuable assets of Earth. Following COP28, bioprospecting is necessary to seek new classes of bioactive metabolites and enzymes for chemoenzymatic synthesis. Focus is placed on those performance and practice modifications which, in a sustainable manner, establish the patient, and the maintenance of their prophylactic and treatment needs, as the priority. Forty initiatives for natural products in healthcare are offered for the patient and the practitioner promoting global action to address issues of sustainability, environmental change, defossilization, quality control, product consistency, and neglected diseases to assure that quality natural medicinal agents will be accessible for future generations.

**Graphical Abstract:**

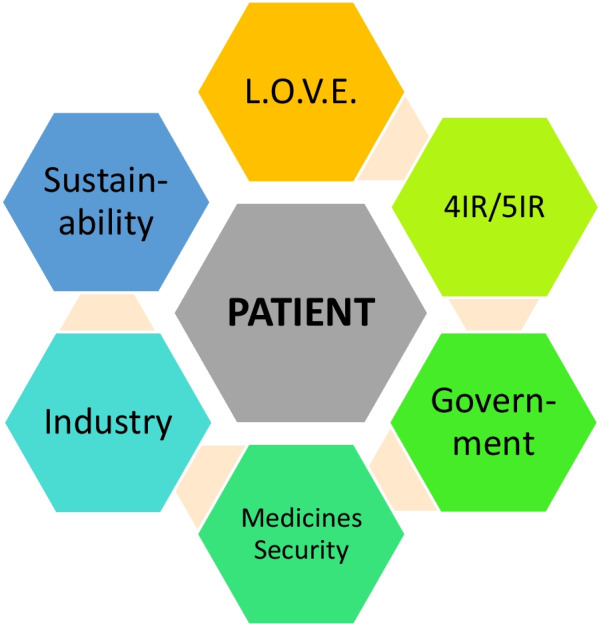

## Introduction

“We are living through climate collapse in real time” according to UN Secretary General António Guteres in his opening statement at the United Nations Climate Change Conference (COP28) held in Dubai. King Charles III indicated at the conference his long-held concerns adding “I pray with all my heart that COP28 will be another critical turning point towards genuine transformational action.” The meeting concluded with, for the first time, a global commitment for countries to be “transitioning away from fossil fuels in energy systems, in a just, orderly and equitable manner…so as to achieve net zero by 2050 in keeping with the science.” There were no timelines, benchmarks, or reporting mechanisms regarding progress established. For organic chemistry, massive implications are apparent for the near future and for ever after. Discussions, tough decisions, paradigm shifts, and aggressive actions are needed urgently, some aspects of which will be discussed in this review as applied to medicines, both natural and synthetic, and their security. A fundamental question for organic, medicinal, and natural product chemists is what is “transitioning” away from fossil-fuel derived chemicals shifting towards?

In an article entitled “Not for us – but for our descendants” prepared over thirty years ago, the author noted that “Preserving indigenous knowledge, developing our resources in a sustainable way, and potentiating the richness of our cultural wealth is the ultimate gift we can offer our descendants” [1]. The thoughts and discussion therein were based on the commentary of the Susquamish Chief Seattle (more correctly Seathl) to whom is frequently, although possibly erroneously [2], attributed the saying “We do not inherit the Earth from our ancestors, we borrow it from our children”. Over 700 years earlier, Saint Hildegard of Bingen (1098–1179), a prolific liturgist, visionary theologist, and the most erudite female naturalist of her time indicated “The Earth which sustains humanity must not be destroyed. For without it, we cannot survive” [3]. We are at a place in human history where that concise, harsh reality must be faced and the potential catastrophic effects of ignoring those truths examined. Challenging, unprecedented lifestyle modifying actions will need to be taken for our endurance, including for those research aspects which currently rely on fossil fuels.

Nature is not a force to be controlled or overcome. We humans are not masters of the universe [4]. We are the transitional keepers, not the owners, of the Earth. It has been stated many times by diverse sources that for humanity to survive our obligation is to work *with* nature to engage a positive outcome. Lovelock saw Earth as Gaia, an integrated, self-regulating biological system of which *Homo sapiens* is only one of the myriad biological components [5]. A detailed discussion and evaluation of this hypothesis based on the accumulated research has been published [6].

Chief Seattle, in his purported letter of response to United States President Franklin Pierce in 1855 [7], placed humans within Earth’s web, indicating that “Man did not weave the web of life he is merely a strand in it. Whatever he does to the web, he does to himself” and presaged the trauma of not respecting Earth, writing “What befalls the Earth, befalls all the sons of Earth” [2]. The letter and its visionary sentiments, even in disputed authenticity, represent a powerful, eloquent, and prophetic warning of which we must now be fully cognizant. Earth and its biospheres are there for humanity to live with, to share with the other millions of organisms, to show reverence and respect for, and, critically, to not abuse our utter dependence on Earths’ resources with our own toxic terrestrial and marine detritus and exploitive behavior.

For thousands of years, the unspoken operational myth regarding natural resources was that they will always be there; until now they are not [8]. It is only in the last 60 years, spurred by more critical assessments of the dramatic decline in Earth’s assets by Rachel Carson, Edward O. Wilson, and many other concerned scientists, that the staggering level of resource destruction Earth is experiencing has become an international cause for concern [9]. E.O. Wilson, for example, referred to Earth as a “Half-Earth”, and proposed a plan to reclaim and protect critical natural habitats for the conservation of species for future generations [10]. He is accurate in his conclusions regarding some contemporary human behavior which debases concerns for planetary health, and which must be overcome for the possibility of compensatory actions to be effective [11]. Only substantive corrective actions, coupled with major paradigm shifts in thought and practice, can avoid a disastrous planetary outcome [12].

## The state of Earth

We, the human population of Earth, as well all other life, are living in the Anthropogenic Era [13], wherein our actions and inactions have had, are having, and will have, profound impacts on many aspects of the biological and environmental systems with which we interact daily are interconnected always. As a result, two factors now overarch all future aspects regarding the investigative optimization of natural resources: sustainability and environmental change. With a global population of more than 8.07 billion (December, 2023), demands for land for arable farming, grazing, and urban development are intense, frequently at the expense of biologically unexplored primary forests in megadiverse countries [14]. Gases (characteristically methane) from grazing animals and thawing permafrost are contributing significantly to climate change [15], together with the emissions from steadily biodegrading accumulated landfills [16]. Environmental change is modulating the growth patterns and availability of essential crops and other natural resources, and their associated pathogens, due to excessive heat, rains, or droughts [17]. Severe weather events are occurring more frequently and in unexpected locations [18]. The massive floods in Pakistan in 2022 which displaced 33 million people are a recent example [19]. Major global cities, such as Jakarta, New Orleans, Miami, Bangkok, Venice, Kolkata, among others, are threatened with almost perpetual or significant flooding over the next 30 years [20, 21].

As the sea levels rise, coastlines are being altered, and a plethora of low-lying islands in the South Pacific [22], such as Tuvalu and Kiribati, and elsewhere in the world are projected by 2050 to be under extreme conditions of inundation with the resulting devastating effects on the fundamental viability of their cultures, housing, food supply systems, and healthcare resources [23–25]. Northern latitudes are also already affected, and in England over 200K coastal homes are threatened to disappear in the next 30 years [26]. This pattern of coastal devastation, discussed for over 45 years [27], will be repeated in many coastal and river estuary environments in the next 50 years [21, 28–30]. The speed at which these changes are occurring has startled climate scientists, especially with the challenges of modulating disease patterns, particularly due to vector-borne diseases [31].

The changing salinity of the soil, as well as coastal erosion, is having an irreversible impact on both the land used for farming and the crops and the medicinal and other commercial plants being cultivated [32, 33]. For half the world’s population rice is *the* dietary staple. However, rising sea levels and the associated salinity and variable weather patterns are devastating rice crops and local economies, as well as depleting nutrient levels, prompting initiatives to find or develop new rice strains that will thrive in the changing environment [34]. Is the food crisis with rice presaging the emerging situation of the unpredictable metabolite modulation of medicinal plants?

Alarms regarding the global threats to humanity have been ringing for more than 30 years. The first “World Scientists’ Warning to Humanity” focused on the unsustainable rates of Anthropogenic devastation to the forests, to biodiversity, to freshwater and the oceans, and to the atmosphere [35]. It called for population stabilization and reduced consumption of non-renewable resources and had 1565 signatories. A second warning, signed by 15,364 scientists from 184 countries, was issued in 2017 and offered additional evidence of the irreplaceable losses of resources on which humanity relies [12]. More recently, a specialized warning focused on climate change and access to medicinal plants was presented [36].

The Paris Agreement of 2015, to which 196 Parties are signatory [37], projects efforts to limit the ambient temperature increase to 1.5 °C, a target which presently seems an impossibility [38, 39], especially given the 425 “carbon bombs” (> 1 gigaton CO_2_ emissions) of fossil fuel extraction around the world [40]. Carbon dioxide, nitrous oxide, and methane levels all reached new high levels in 2022 [19]. The Chair of the Intergovernmental Panel on Climate Change report of February 2022 Dr. Lee Hoesung indicated that the report “shows that climate change is a grave and mounting threat to our well-being and a healthy planet. Our actions today will shape how people adapt and nature responds to increasing climate risks.” He continued by indicating that the report “emphasizes the urgency of immediate and more ambitious action to address climate risks. Half measures are no longer an option.” [41] The year 2023 is established as being the hottest on record with each month since June hotter than the corresponding month in previous years [42]. Our descendants will rightly blame the generations of the middle-late 20th and early twenty-first centuries for their inaction and for the resulting, drastically modified, quality of life if we are not sufficiently responsive.

At this juncture, it is appropriate to introduce an acronym to bring focus, context, and enhanced awareness to some of the selected topics for consideration. That acronym is **L**.**O**.**V**.**E**. and it represents **L**earning to **O**ptimize the **V**aluables of **E**arth. Inherently, humans have continuously pursued the selection of optimum naturally sourced materials for their use, the strongest woods, the most robust crops, the most pungent spices and fragrant oils, the best places to live and be productive, and the most effective agents for healing, for the amelioration of pain, and as biocides, and numerous other uses resulting in their successful naturalization [43]. Those past optimizations for healing evolved through extensive random trial and error experimentation and were related typically to a specific, timely use with little consideration of regeneration or replenishment because the local need was limited, based on population, compared with the available resource. The evolved paradigm (actually a myth!) was that the natural materials would always be there, available, and ready to use, even if cultivation and selective forestry was occasionally needed. In addition, fossil fuels have underpinned many of these activities through the chemicals derived from oil and coal. Those paradigms, over the next years will shift, for humankind must optimize resources (volume and space) for long-term availability, not instantaneous utilization and disposal. Thus, bioprospecting will assume new challenges for discovery from diversified sourcing and for a new phalanx of intended outcomes to support the lifestyle of the future generations as the uses of fossilized resources will need to be replaced by innovative, sustainable alternatives (vide infra).

## What’s on Earth?

In this Anthropogenic Era [13], assessing and documenting the existence of the remaining organisms of Earth, prior to investigation, has developed as a race against time. Only those existing and accessible plant (and other) species can be catalogued, even as newly identified species are themselves facing extinction. It is impossible to fully assess what species have been lost in the past years of the Industrial Revolution (IR), and that retrospective, while a useful consideration, can distract from what presently exists and the conservation initiatives needed for future generations [44]. In September 2020, the Royal Botanic Gardens Kew issued its fourth *State of the World’s Plants and Fungi*, a collaboration of 210 researchers in 42 countries [45]. The Plant List maintained by Kew accepts 350,699 plant species names in 642 plant families harboring 17,020 plant genera. Of these species, 25,791 (7.35%) are plants with a documented medicinal use, and 7039 (2.00%) plants are recognized as being edible species, although only 417 are regarded as crops. Of the medicinal plants, 541 are on the International Union for the Conservation of Nature Red List and 723 are threatened with extinction, and of the edible plants, 30% are on the Red List, and 234 are threatened with extinction. More than 30,000 plant species are protected under the Convention on International Trade in Endangered Species of Wild Fauna and Flora [46]. How are those 957 plants threatened with extinction to be considered for the future? Are they all to be conserved? Is that justified based on existing scientific evidence which suggests a potential use as a medicine or a foodstuff? How are priorities to be established? Without a possible relevance to human development, is being an integral and interconnected facet of the “web of life”, probably for other species in Gaia, an adequate rationale for saving them? Or does “humanity” consciously let them disappear? Now and forever, humanity is faced with the excruciating dilemma of how to create a balance between technological progress and maintaining the assets of Earth for future bioprospecting [1, 47].

Between 1998 and 2013 the number of medicinal plants in commerce dropped dramatically from 700 to 350. This possibly reflects sustainability concerns and/or diminution of access (i.e., disappearance from a local habitat through overharvesting) [45]. It is well-recognized that the collection of bark, bulbs, and root materials from wild-crafted species for medicinal purposes, or for drug discovery programs, is especially destructive to the future sustainability of medicinal plant species. In 2019, botanists registered 1942 newly identified plant species and mycologists identified 1886 new fungi [45]. Will any of those 3828 species contribute in the future to sustainable human development in terms of food, medicine, biofuels, essential oils, insecticides, shelter, cosmetics, and other purposes yet to be identified? For the late naturalist E.O. Wilson, Earth remained “a little-known planet”, with probably less than a fifth of the overall biodiversity catalogued [10]. How can we implement L.O.V.E. and pursue the correlative bioprospecting initiatives in various desired directions (e.g., neglected diseases) without knowing what organisms might be available as a food crop or as a health or agricultural beneficial entity? How do we assess global availability and thus what organisms should be maintained as sustainable resources, *before* they are gone, forever?

Preservation of genetic resources for possible regenerative purposes occurs through seed banks and plantations established in 350 botanical gardens in 74 countries. In addition, there are 3324 herbaria in the world with 392,353,689 specimens as of 12/2019. As noted, linking the information on these acquisitions is an on-going artificial intelligence (AI) project, but based on historical acquisitions and colonialist activities and mindset, there are vast imbalances and functional gaps in the locations of herbaria. For example, the island of New Guinea has 13,634 known species, with only five herbaria. In contrast, the United Kingdom has 2,233 native species and 223 herbaria [45]. Approximately 31% (107,340) of the known vascular plant species are being grown in botanic gardens, although 93% of those gardens are in the temperate regions. From the perspective of fungi, there are an estimated 2.2–3.8 million species, although only 148,000 are thus far named, and only 17% of these (25,611) are cultured and available. There are 793 fungal culture collections in the world in 77 countries, however, the whole of the African continent has only 18 fungal collections [45]. There is much to be accomplished in training and infrastructure development to foster new botanic gardens, seed banks, herbaria resources, and the acquisition and genomic identification of fungi from the diversity of their locations.

As indicated by Wilson, explorations for a sustainable Earth are highly relevant for the world population at this time of dramatic environmental change [10]. However, the NASA budget for 2023 is about five times that of the whole of the World Health Organization (WHO) (US$ 32.35 bn vs. 6.725 bn) and ten times that of the Food and Agricultural Organization (US$ 3.25 bn). Realignment of investment (i.e., a paradigm shift) in applying L.O.V.E. to optimize the known edible and medicinal resources in a sustainable manner and exploring new resources to meet existing and anticipated challenges facing food and medicine supplies, should be a high priority, global activity. This requires a focus on future patient needs for all medicinal agents, natural and synthetic, embracing comprehensively the concept of “Medicines Security” (vide infra) [48, 49], as defossilization begins to impact chemical and natural resource supply chains.

## Balancing Earth

It is an indisputable precept that “The first law of the natural world……is interdependence” [4]. As a consequence, the reliance of humanity on the gifts from Earth is unshakeable and sacrosanct. It is also axiomatic that the health, indeed survival, of the world population is inseparable from the health of the planet itself [50]. The natural world that we call Earth is our sole source of shelter, of furnishings, of clothes, of food crops, of spices, of biological agents, of cosmetics, and of healthcare resources. There is no plan(et) B. After 4.5 billion years of Earth’s evolution, the fate of the planet and its “life” is now in the hands of a very few members of *H. sapiens*. *Sapiens* notably, was derived by Linnaeus from the Latin verb *sapere*, meaning “to be wise”. It is the wisdom of broad and meaningful local and global actions that will be necessary as we contemplate and redefine the future of humanity on Earth. That will necessitate healing the Earth and healing ourselves into a different conscious space. Focusing the discussions (and actions!) regarding environmental change, biodiversity and species losses, population growth, food security, medicines security, pollution, and habitat changes in the coral reefs and the mountain climes must engage the fundamental precepts of Chief Seattle and Gaia…. Namely, the inextricable interconnectedness of nature, including humans, that has developed over the past 4.5 billion years. How did we get so rapidly to this dramatic point in Earth’s evolution?

What has occurred on Earth from the 1st to the 4th Industrial Revolutions (1760-present), is that society, especially the Global North, has neglected to embrace that core premise of interdependence with nature. As a result, the successive technological advances have brought us to the global chaos of dramatic environmental change (flood, fire, drought) currently being witnessed first-hand, typically unexpectedly, by geographically diverse communities almost every week. Under these rapidly changing environmental conditions, for the future of natural resources and their multifactorial role in society to be maintained and expanded, particularly regarding healthcare, paradigms must be torn down, gaps must be filled, new alliances forged, new actions initiated, and new, ethical contracts established. Awareness and well-funded leadership actions for the necessary conservation of Earth is essential. Without this commitment to contemporary action, what will future generations have to work with? A wide range of suggestions for specific action areas to be promoted to modulate the paradigms of traditional (TM) and complementary (CM) medicine are presented subsequently.

## Sustainable interactions with nature

Almost every aspect of our daily lives depends on an interaction with naturally resourced materials. All the compounds utilized in the plethora of consumer goods and foods, and for biological and medicinal purposes, arise from Nature through previous efforts at bioprospecting over the millennia. Some products are available directly, the so-called natural products being enzymatically produced each moment by the diversity of Earth’s organisms. Others have been produced historically over eons indirectly through extensive chemical reactions on the non-replaceable, fossilized products of coal and oil. The availability and biological effects of this compound diversity are at the core of the food and medicines which support a healthy human life, as well as the lives of every organism, sometimes positively, other times not so. It is vitally important that all chemical and biological scientists become regenerative environmentalists, showing deep reverence for Nature, to maintain, extend the lifetime, and, where possible, regenerate the Earth with shrubs and trees to assure the availability of those resources for our descendants. It is essential to grasp that sustainability *is* now *the* journey for all humanity on Earth for as long as we humans exist [51].

A different and rapidly evolving and relatively new economic factor of sustainable development is presently in play as countries experience a real, annual *loss* of population [52, 53]. Many European countries, together with Korea, Japan, and China are witnessing low birth rates, where governments, and even Pope Francis [54], are encouraging families to have more children rather than pets. Ageing populations, because of improved health care, and low birth rates, and in part due to the high costs of marriage and child-rearing, ultimately strain latter-stage medical resources. The prediction is that there will be insufficient adult earners to fund the required tax base for the national healthcare systems for the aged, with Japan as a model system [55]. Incentivized efforts to continually enhance Earth’s population are not a sustainable pathway to either a healthy or a harmonious planet. Such programs will undoubtedly lead to the accelerating loss of natural, non-regenerative resources, and a significant increase in economic hardships and international social injustice [56]. Succinctly, as Jack Welch, former Chief Executive Officer of the General Electric Company indicated, there is an orphic need to “Face reality at it is, not as it was or as you wish it to be.” The implication being to plan accordingly. It is those plans for future needs with respect to aging populations and modulating disease prevalence which global natural products research needs to act on through our contributions of highly specialized experience and knowledge.

“Green” chemistry [57], with its extension into natural products chemistry through “ecopharmacognosy” [58], has resulted in new dimensions for how processes (reactions, extractions, separations, metabolite characterizations, and preliminary biological assessments) are conducted. It also addresses “performance” concerns, and how research laboratories and industrial production sites, can change their day-to-day philosophies and practices [47, 48, 58–62] to apply greater focus on benefitting humanity in a manner that is both sustainable and regenerative. These attributes are, and will continue to be, critical elements for the applications of natural products as transitions and replacement strategies begin to occur. Reductionist bioprospecting approaches to drug discovery for example, frequently focus on an effective function with minimal other considerations, such as product biodegradability. Ecopharmacognosy specifically and inherently associates the specific biological function with the added, upfront “performance” requirement of sustainability and inherently, biodegradability [47, 48, 58–62]; a subsequent feature to the pioneering discussions of “green” chemistry [57]. Such environmental considerations must be included as an integral aspect of the fit-for-purpose approval of all regulated chemical entities and complex natural product matrices, as well as being included in the development of innovative regenerative and (bio)circular economy research programs.

For foods and medicinal agents, irrespective of source, in a time of environmental change, continuing availability of quality assessed products is critical. Additionally, eliminating, repurposing, or at least minimizing the organic waste generated through industrial processing is evolving as a priority for a sustainable future. During most of the past 160 years, the world has operated on a linear economy approach of almost unfettered expansion based on a dramatically increasing and ageing population which creates and supports enhanced technology, globalization, and advanced consumerism with somewhat minimal regard for the contemporary or long-term environmental impacts, including waste processing and biodegradability [63]. Currently, it is realized that this paradigm is transitional, and that for the future, economies must be based on sustainability, not unlimited population growth or the differentially regulated exploitation of Earth’s resources [4]. However, “Sustainability requires living within the regenerative capacity of the biosphere” [64]; a capacity which likely has already been exceeded [65]. Efforts to move to a circular (bio)economy, in which continuous resource depletion is uncoupled from economic growth and waste, is now valued for its investigative potential [63], and are a prime example of contemporary L.O.V.E. Yet, for that paradigm shift to be effective globally, and beyond a few isolated economic systems, significantly enhanced bioprospecting initiatives and investment will be required for the development of biorefineries, as transferable, cost-effective systems, so that organic waste can be converted effectively into useful chemicals, fuels, and bioenergy as a matter of priority, and, eventually, necessity.

Given that the journey of sustainability is now an fundamental core wisdom for a quality life on Earth for *every* future generation; unlimited economic growth is simply not a sustainable option [4]. Sustaining life for future generations means doing less with new resources and doing more, creatively, with pre-used, bio- and other resources (colloquially termed “waste”). Even a massive regenerative mindset and action pathway cannot undo the damage from the successive four/five industrial revolutions. Indeed, that tipping point is now only of historical interest [49]. Thus, accessibility, inexorably and intimately, applies to the sustainable development of all natural products used in patient care in addition to TMs/CMs, particularly those important regimens requiring numerous, non-cultivated plant constituents [4]. The burgeoning Fifth Industrial Revolution (Industry 5.0; 5IR) must provide the investigative opportunities for paradigm shifting towards natural resource optimization (vide infra) [8].

The sustainable aspects of medicines particularly those derived from nature with a historical pattern of use, represent critical, albeit undervalued, core knowledge regarding planetary and therefore human health. As indicated many years ago, “It is time for the health care industry to think in terms of medicinal agents as a sustainable commodity which, in their provision to the global population, does not deplete the resources of the planet” [49]. That admonition applies to synthetic, semisynthetic, and natural product medicinal and other biological agents. Investments by governments, industries, and international agencies in healthcare development for the patient must promote and maintain a harmonious relationship with nature, with the community, and with all the stakeholders. As an integral component of assuring availability within Medicines Security (vide infra), industry will need to place a focus on a detailed assessment of the sustainability of the source materials for medicinal agents, and an appreciation of their environmental impact and biodegradability. Absent those considerations, the long-term access to the current (and future!) synthetic and nature-derived medicinal agents will, inevitably, be jeopardized worldwide, irrespective of the overall economic and functional status of the local healthcare system.

## Medicines security

For the patient, the necessity for transformative paradigm change is embraced within the concept of “Medicines Security” which applies to all medicinal agents, irrespective of their origin, synthetic or natural [47, 48, 61, 66]. “Security” in this sense covers four essential elements. The first is the sustainability of the source, be that natural from a plant, an animal, or a microorganism, through a series of synthetic steps of fossil fuel origins, or a combination of the two processes. The second is the scientifically assured integrity of the standards of practice for the quality control, acquisition, manufacturing, stability, storage, and delivery of the product to the patient. The third is maintaining the accessibility to approved medicinal agents, natural and synthetic, i.e., that the respective preparation will be both available and affordable when needed by the patient, especially when the utilization is for a chronic health need. This is of particular concern with respect to the vast unmet needs for orphan drugs which are typically based on non-renewable resources [67]. Currently, for selected cancer chemotherapeutics, a very high percentage of major cancer centers in the United States are experiencing drug shortages of important therapeutic agents such as cisplatin and carboplatin [68, 69], as well as the podophyllotoxin-derived, etoposide [70], where decisions due to drug rationing in hospital systems become a life-or-death choice [71]. More widely, shortages of crucial pediatric antibiotics, such as amoxicillin, are now occurring [72]. Assessed in the United States Senate as a national security threat [70], these long-term shortages are also viewed as occurring in a continuously recycling mode unless modifications the persistent issues of quality sourcing within international supply chain systems are abrogated [73].

The fourth aspect of Medicines Security is the security, in Industry 5.0 terms, the resilience, for the patient and the practitioner, of maintaining the quality, safety, and unequivocal, demonstrated authenticity of the product and the potential healing or prophylaxis that the patient inherently seeks for the acquired medicinal agent. This occurs through the establishment, application, and enforcement of local and international standards by assessment using contemporary integrated technologies such as blockchain [48, 62, 66]. In other words, that the product is presented at an approved full-strength, is not adulterated, contaminated, or substituted, and has an established safety, efficacy, and stability profile providing reproducible bioactivity. In addition to the health concerns for the patient for prescription products, there is a fundamental ethical disconnect regarding the oath of practice for practitioners and pharmacists who recommend a traditional or complementary medicine with no assurance of quality at the purchase site (retail store or on-line), and consequently that *their* anticipated health benefit for the patient is capricious [74].

In the twenty-first century, it is indeed a shameful commentary that the fundamental attributes of good collection, agricultural, manufacturing, and standardization practices, and the supply chain and product timelines are questionably regulated across the globe. The Medicines Security sought by the patient is inherently linked with the previously discussed concept of the quality, safety, efficacy, consistency in content, and accessibility ( availability and affordability); summarized as Q.S.E.C.A. [47, 48, 58, 59, 75]. Distressingly, the absence of adulteration, or of potentially toxic contamination, is not legally or analytically assured daily, for the billions of patients with respect to nature-derived products. Indeed, the facile accessibility of ordering medicinal agents through unregulated, on-line supply chains has worsened this situation for patients from the perspectives of the quality and content of the marketing information and of the product sold [76]. In general terms, for the evolution of traditional and complementary medicines to become patient-centered, approved, and acceptable in integrated healthcare systems, securing all the feasible evidence to treat you (safety), the patient, as well as acquiring all the essential facts and figures indicating the clinical appropriateness of the chemicals for you (efficacy). For patient benefit, the products must meet those evidentiary standards and ethical requirements on a consistent basis. For the government regulatory authorities, collaboration with industry and academia is essential to establish and enforce those standards.

## Natural products in healthcare

The demand for medicinal agents continues to increase dramatically [77]. In the past 63 years the population of Earth has risen from 3.03 billion to 8.07 billion (December 2023) and will reach 9 billion by 2037 [78]. Life expectancy in the United States was 48.2 years in 1900, whereas in 2020 it was 78.8 years, and worldwide it continues to increase as food and sanitation standards and access to prophylactic and therapeutic regimens improve [79]. Based on an ageing population, who have a distinctly higher use for medicinal agents, although some of it may be inappropriate [80], the non-renewable resource implications for this ongoing process of globally expanding ageing are staggering [10]. International agencies are charged with oversight of these stunning transformative health and social outcomes, and with making recommendations for their food management for the diverse communities of Earth, but, stunningly, not for the requisite medicinal agents (vide infra).

The Constitution of the World Health Organization (WHO) dates to July 22, 1946 and has been ratified on several occasions in the intervening years [81]. One of the fundamental principles of the WHO is “The enjoyment of the highest attainable standard of health is one of the fundamental rights of every human being without distinction of race, religion, political belief, economic or social condition.” In addition, one of the twenty-two committed functions of the WHO (Chapter II, Article 2, u) is: “to develop, establish and promote international standards with respect to food, biological, pharmaceutical and similar products”.

Furthermore, Article 21 of the Constitution indicates that the WHO Health Assembly can adopt regulations concerning: (i) standards with respect to the safety, purity and potency of biological, pharmaceutical, and similar products moving in international commerce, and (ii) advertising and labelling of biological, pharmaceutical, and similar products moving in international commerce [81]. The power to make a dramatic paradigm shift regarding recommendations for the quality of commercially produced traditional medicines and to enhance patient care is therefore available; however, simply put, it is not being pursued aggressively enough to place patient benefit as the highest priority; a paradigm shift is called for.

The most recent compilation of WHO Health Statistics for 2022 [82] summarizes key details of healthcare performance in the 194 Member States responding to the data analysis regarding 50 aspects of the Sustainable Development Goals (SDGs) [83]. There is limited discussion in the report of the need for integrated healthcare, and the desire for more bioprospecting effort to be placed on neglected tropical diseases is mentioned sixteen times. However, from the perspective of those medicines which serve the developed and the less-developed worlds, the statistical analysis is woefully inadequate. There is for example, no mention of the introduction of newly approved drugs by Member States, and no mention of the regulations for the quality control of medicines and their analysis, which are fundamental aspects of SDGs 3, 10, 13, 14, and 15 [83]. Stunningly, the terms “traditional medicine” or “herbal medicine”, the primary healthcare practices of most of the world population, do not appear anywhere in the report. The substantive aspects of “Medicines Security” relating to an assessment of the continued availability of approved medicines for the benefit of the patient are not mentioned. These are substantial deficits which require consistent assessment, with subsequent accountability and remediation, to effectively monitor healthcare performance.

A wide range of new natural products and their derivatives have contributed to drug regimens that provide United States Food and Drug Administration-approved treatments and curatives in the past 40 years [84]. Importantly, these efforts continue to be successful, encouraging the continued exploration of available natural resources, whether terrestrial plants, marine organisms, or diversely acquired microbial organisms, and based on a variety of considered alternative strategies [85]. Raising awareness regarding local environmental issues, and examining and potentiating local resources to benefit humankind, including the continuing search for new and sustainable natural medicinal and biological agents, are some of the most important contributions that natural products researchers in various countries can make. Such initiatives can address local healthcare needs for the provision of hit metabolites through the evolving strategies for drug discovery which other discovery avenues do not address [86, 87].

The importance of traditional medicines in healthcare systems around the world is poorly acknowledged and appreciated financially, even though it is *the* basis of healing for most of the world’s population and has been an invaluable resource for drug discovery and development. There are some exceptions from an integrated, medicinal agent systems perspective, notably China, India, Japan, Myanmar, and Korea. Whether one looks internationally at the World Health Organization, or by a country such as the United States, or the United Kingdom, or countries in South America, or most of South and Southeast Asia, the research programs on traditional medicines and phytotherapeuticals have been poorly funded as a priority, are typically random in approach, and are not systematically prioritized based on prevalent patient disease needs. Investigations of non-sustainable allopathic medicines, especially for chronic diseases, have consistently held a significantly higher priority, despite the dire need for new antibiotics and other agents to counter new challenging organisms and multiple forms of drug resistance [88], as well as initiating discovery programs for neglected diseases where drugs do not exist or are toxic [87, 89, 90]. That paradigm must change, for two prime reasons, as discussed, many years ago, regarding a holistic view of natural products in global healthcare [48, 59, 62, 86, 91], namely, access to safe and effective agents for the breadth of global disease needs and their secured quality control to “mind” health gaps.

Without continuing access to the natural resources for traditional medicine, health care systems worldwide would be severely diminished, as importation costs for desired synthetic drugs would soar in the marketplace. Medicinal plants, in addition to functioning as prescription or over-the-counter products, as traditional medicines, as complementary medicines, as prophylactics, or as well-being enhancers, are an integral component of the polypharmacy practices of patients globally, particularly those in developed countries [92]. Therefore, maintaining access to sustainable medicinal plants and establishing on-going, broad-based, and well-coordinated bioprospecting of prioritized medicinal plants, supported by the inclusion of machine learning (ML) algorithms for extensive information systems, is a very important activity to support a healthy population. These programs should be highly collaborative and based on the Triple Helix development model of functional collaborations between academia, government, and industry with patient benefit as the priority. In some areas of the world, regional collaborations to optimize available resources will be necessary to achieve functionality. At the same time, it must be acknowledged that certainly for the 25,791 medicinal plants thus far reported, or even the 350 medicinal plants that were prevalent in commerce as of 2013 [45], establishing quality, safety, and efficacy for *all* the potential products is a daunting, likely impossible, task. A rational prioritization for medicinal plant research will therefore be essential.

From a patient and a practitioner perspective, medicinal plants and their regulation are inextricably intertwined as fundamental elements in any larger vision for integrated health care systems. Integration of practices requires mutual respect based in evidentiary science. Inherently, within a reductionist model, the establishment of safety, efficacy, and consistency of a well-defined preparation are core aspects of ethical medical practice. However, in the absence of philosophical change and practical initiatives for safety and functional evidence, the long-term prospects for healthcare integration are dim [48, 62]. Subsequently, this presentation will identify forty broad-based initiatives to enhance traditional medicine practices and products with patient benefit as the highest priority, and a more integrated healthcare system as a potential outcome. The goal, in the Anthropogenic Era [13], is to achieve a higher level of Medicines Security [47, 48, 62] through a deeper understanding of the multiple factors involved as the dire threats of environmental change, loss of biodiversity, and “transitioning” from the dominant fossil fuel base, impact in a profound manner global health care discovery and delivery.

## Natural products are a key to global health

Although the global pharmaceutical manufacturers would have patients believe otherwise, synthetic, fossil-fuel-derived, pharmaceutical agents are, for most of the population of the world, not the key to their health, and nor will they ever be, as supply chains are inadequate based on need and volume, and the required chemical resources are being depleted continuously resulting in medicine shortages (vide infra). Many products are simply not accessible; either they are not available locally, or they are too costly, unless local production or biosimilars can be prepared [93]. Emerging economies at various levels cannot afford to import the higher cost synthetic drugs or biologics currently available in the Global North and many patients in low-income countries must rely on local, typically unregulated, TM markets. It is one of the many areas where healthcare worldwide is out-of-balance with patient needs [94]. An international consortium is needed to take responsibility to promote a higher level of patient care for this situation. Governments in emerging countries, as well as in many developed countries which are not major pharmaceutical manufacturers, practitioners and their patients are continuously at the mercy of the major pharmaceutical and generic manufacturing companies, particularly when the cost of a particular drug may vary many-fold, depending on the country [95]. There can be no Medicines Security without a major shift in this paradigm towards focusing collaborative research on local healthcare needs which are established through examining the healthcare “gaps” in the functional coverage for the patients, and based on sustainable natural resources [86, 96].

For the fundamental drugs of international commerce there is a lack of manufacturing and distribution capacity to make approved drugs more widely available [97]. The chasm is enormous, even in developed countries which lack a national health care system, such as the United States. A drug-based therapeutic intervention by a health practitioner assumes accessibility (availability and affordability) to the desired regimen. The United States Food and Drug Administration lists over 250 drugs (typically) which are approved although not available for physicians to prescribe for patients [98]. In addition to international supply chain failures for finished products, access by manufacturers to key chemical ingredients and production costs are evolving where it is no longer profitable to make a particular drug. As a result, the “security” of accessibility of approved and effective medicines, including front-line anticancer therapeutic agents [68], has been lost and the patient suffers, literally. As discussed elsewhere in this paper, the relentless depletion of core chemical resources from oil and coal will likely exacerbate this situation, in the absence of urgent corrective measures.

Quite separate to these concerns, which focus on approved drugs meeting the chronic needs of cancer patients, where rationing is occurring in the United States [70], for those patients with chronic conditions or for adjuvant of prophylactic use, there is the chasm of drug discovery. To address the profound discovery gaps in global healthcare, one important paradigm must be dispelled, that patients are only relevant when they are pharmaceutical profit centers for cancer, heart disease, neurological diseases, diabetes, obesity, and several vector-borne diseases, i.e., the chronic and lifestyle-induced diseases of humanity [99].

Reversing that paradigm would put the prophylactic and treatment aspects of medicine development for the patient first, on a worldwide basis. According to WHO, and prior to the Covid-SARS2 pandemic, an estimated 2 billion people (25% of the global population) did not have access even to the essential medicines of the WHO list [100]. In 28% of Member States no medical facility in the country provided access to all the recommended essential medicines [101]. Who are the stakeholders with the resources and the passion and the ethical standards to participate financially and scientifically to discover and develop new therapeutic agents for the myriad of diseases for which a medicinal agent has yet to be identified? In terms of clinical treatment, analysis of the World Statistics 2022 report reveals that in 2020, 22.2% of the global population required interventions relating to neglected tropical diseases [82]. Forty-two of the 194 Member States each had over 5 million interventions for neglected tropical diseases. For how many of these diseases is any focused, strategized drug development pipeline based on sustainable sourcing underway utilizing the advanced AI/ML-directed resources of the major pharmaceutical companies around the world? Where is the compassionate action for a patient population in desperate need of effective medicinal agents?

International investment in pharmaceutical drug development is skewed towards the healthcare needs of about 10% of the global population. Every low-income country in the world has > 5 neglected tropical diseases simultaneously prevalent [102]. However, even in this situation, research on the 20 WHO-recognized neglected tropical diseases fares very poorly [103] and merits, indeed necessitates, a much higher level of priority for sustainable drug discovery [104]. Exemplifying this reprehensible situation, very few new chemical entities were approved worldwide from 1975 to 2022 for tropical diseases [84]. In addition to supporting massive drug administration programs of existing drugs [104], with the attendant concerns of drug resistance [105], some pharmaceutical companies (Glaxo, Novartis, Sanofi) have initiated academic/private institution research partnerships. However, these typically do not develop local infrastructure and expertise which would provide for continued drug discovery exploration in the emerging world. In addition, the COVID-19 pandemic has pulled significant funding away from neglected tropical disease research [106]. As indicated previously [86], “New international initiatives…. should develop a more structured, well-funded, broader approach that reflects evidence-based traditional medicines. Programs must also build commercial capacity and develop appropriate protections for intellectual property rights.” Also, and pointed out many years earlier, “It should be apparent to anyone interested in long-term health on a global basis that *all* scientifically rational and economically feasible approaches should be employed…” [107]. That is a fundamental aspect of bioprospecting those “valuables of Earth” which expressly include optimizing ethnomedical and ethnobotanical information on the use of natural resources with the contemporary applications of algorithms from AI/ML systems. The need for action is profound, and the gaps to be filled are vast, even at the highest international levels, where the paradigm of pharmaceutical company dominance in prioritizing profitable research outcomes discounts desperate global healthcare needs.

## Quality of natural product preparations

From the perspective of medicines, vaccines, biologics, and diagnostics, but inexplicably not TM/CMs, the Oxford Statement on the global quality of medicines expressed deep concerns [108]. It called for action to provide quality-assured medical products on behalf of patients by supporting World Health Assembly Resolution 67.20 regarding the strengthening of national regulatory authorities. It defined critical initiatives for improvements in the prevention, detection (laboratory and in-field), response (product removal), and education domains [108]. It called for investment, policy change, and action to eliminate substandard and falsified medical products. In terms of TM/CMs these changes are fully embraced by Q.S.E.C.A., as discussed previously [47, 48, 58, 59, 62, 75].

Although many regulatory, and even pharmacopeial, systems would have scientists, producers, manufacturers, practitioners, and patients believe otherwise, the fact is that as a Latin binomial “a plant is not a plant”. Accurate macroscopic and DNA-based binomial taxonomic identification does *not* assure a quantitatively reproducible metabolomic profile for the biologically significant metabolites. This is a fundamental requisite for the quality control of biological consistency, the trusted and anticipated element for all patients. Other factors, including adulteration (additional plants or synthetics), contamination (pesticides, heavy metals, microbials), and substitution (different plant or plant part), render the name *on* the box dissociated from, and in practice irrelevant to, the content *in* the box (vide infra) [62].

All living organisms, including their endophytes, epiphytes, and symbionts, produce a plethora of metabolites in various chemical classes, based on a surprisingly limited number of precursors [109]. Yet, as the environment changes through access to water, soil pH, microbial rhizosphere diversity, heat, cold, light, altitude, moisture, so the regulation of the genes producing the characteristic constituent metabolites, in plants, marine organisms, and microbes, will be modified arbitrarily, and, at present, uncontrollably. As a result, the biological outcomes produced by that organism will be altered, as observed in alpine plants [110]. For plants which serve as nutritional food crops or as sources of medicinal agents, the impact on the metabolome may be extensive, either potentiating or diminishing the bioactive metabolites of interest [32, 111].

Metabolite profile modulations were not predictable when considering environmental temperature changes for the growth of *Arnica* species [112], for isoflavonoid levels in soybeans [113], for the yields in oilseed crops [114, 115], for carotenoid levels in tomatoes [116], as well as the impacts of drought stress [117, 118], including on culinary herbs such as sweet basil (*Ocimum basilicum* L.) [119]. Biomass volume will also change, leading to possibly lower yields of a crop, but higher yields of active constituents, thereby modulating the effective dosage [120]. This outcome will also cause economic volatility in local markets for the harvesters. Like ex situ conservation efforts, due consideration is needed to assure that these impacts do not further diminish the access to medicinal agents for isolated communities with existing limited healthcare options and/or economic resources [36]. It is a further example that a regulation applied to a plant extract requires significantly more scientific guard rails than a Latin binomial and a plant part, and should reflect the application of contemporary technology.

The implication, from a regulatory or pharmacopeial perspective, is that the assessed and specified concentration range for bioactivity may quickly lose its relevance under different growth conditions, including those induced by climate change. To maintain the nutritional value of the crops, and the medicinal value of the TMs, detailed assessment of these metabolite changes is urgently needed to assure consistency in the anticipated nutritional and/or medicinal benefits for the consumer/patient [48, 58, 59, 75]. As a result of environmental modulations, the next 20–30 years will also see unprecedented shifts in the in-field/forest locations of medicinal plants as environmental change intensifies in many areas of the world where the security of accessibility is essential [121–124]. The paradigm of consistent availability (a medicines security) for plant recollection in a specific, previously documented, area will disappear, as new growing areas are identified, concurrently impacting the metabolome and the market price [125].

It is well-established that the ranges of some plants, including medicinal plants, are moving to more favorable growth environments due to climate change, a phenomenon being observed and modeled in several mountainous areas [126–130], reflecting the plants’ need for the cooler temperatures offered at higher altitudes [131–134]. These changes may also impact the web of interconnectedness of the existing flora and fauna causing fragmentation of species distribution and challenges for pollinating insects and grazing fauna [135–137]. In other instances, enhanced drought conditions are altering medicinal plant crop yields, with *Glycyrrhiza uralensis* Fisch. ex DC. (Fabaceae) as an example [138]. In its’ desert steppe habitat in the northern autonomous regions of China it has become endangered and is now a protected species [139, 140]. Formerly, China was an exporter of the roots of *G. uralensis*, whereas now it is an importer of this highly valued medicinal plant [36]. The increased prevalence of harmful insects, such as bark beetles, due to a warmer environment are also causing significant damage to ancient coniferous forests [141, 142]. As projected many years ago [143], climate change is also increasing vector-borne human disease transmission [144–146], extending the development period for mosquitoes which enhances the prevalence of malaria and challenges the possibility of local eradication [147–150]. The return of malaria to Florida [151] is an example. Climate modulation is also having a significant impact on the lifestyles and lore of indigenous groups in many areas of the world [152–155].

Indonesia is one of the 17 mega-diverse countries, and a Government-funded study may serve as an interesting model, with some reservations, as to the conservation needs for medicinal plants. The conclusions were derived from a detailed, survey-based modeling analysis of the projected distribution throughout the vast expanse of its 6,000 inhabited islands [156]. One conclusion was that over half of the populations of the medicinal plants will lose up to 80% of the area of their distribution, in line with other studies [157]. The outcomes will include an increase in species in the Vulnerable and Endangered categories in Indonesia on the Red List, as well as a gain in species populations where new areas for distribution are possible, although only a third of the medicinal plant species would be in the latter category. Twenty plant species were identified to be prioritized for both in situ and ex situ conservation efforts. The missing factor in the study was a relationship between medicinal plant usage in terms of volume (i.e., long term sustainability) and purpose, specifically with respect to maintaining and addressing practitioner and patient care needs. It is important to restate that the tipping point of maintaining all threatened and endangered species is long past; a paradigm shift to maintaining essential medicinal plants for future optimization is now required. Characteristics for importance based on scientific evidence of local and/or global use must be developed through well-funded biodiversity and safety and efficacy investment programs. Sentinella et al. [158] identified that over 50% of tropical species have a germination rate that, because of environmental change, is declining, whereas 95% of species growing at latitudes above 45° will benefit from environmental warming.

With the plant material on hand let us turn to the content for the benefit of the patient. In 1599, Shakespeare, in *The Most Excellent and Lamentable Tragedy of Romeo and Juliet*, summarized succinctly the present imbroglio in TM/CM product quality control with the eponymous phrase, “What’s in a name? A rose by any other name would smell as sweet.” The name doesn’t matter, only the content does. The patient is anticipating a benefit from an assured, reliable content, as Juliet’s love is based on the inner characteristics of Romeo, not his last name. Regulations for TMs/CMs frequently focus on the organism’s name and what can and cannot be stated and claimed *on* the package from a health beneficent perspective. Standards and regulations for what is *in* the package, what the patient *actually* experiences from a clinical medicine perspective, are minimized. This represents a grand and obsequious diversion and abrogates for the manufacturing industry the profound ethical and moral responsibilities for ensuring product safety and reproducible functionality for the patient. When the respective national government does not enact appropriate and implement strong regulatory measures to assure safety, efficacy, and consistency through product surveillance, they too are complicit in this moral morass. The self-regulated marketplace, what the practitioner and the patient experiences online and in the retail store, is consequently a product nightmare, everywhere. A situation where the prevailing uncertainty of dubious substandard products [108] and diminished trust from practitioners and patients [159–161] continuously engenders *caveat emptor* for the consistent benefit of the manufacturer [162–164].

Fraud in the commercial delivery of medicinal agents is rampant globally. The WHO has indicated that for developing countries, at least 10% of all medicines, allopathic and traditional, are inauthentic [165]. However, their data sources were based on the random reporting of a small sample size over several years, and were not part a Member State-based, systematic, structured survey, and consequently are probably a gross underestimate. When DNA-based methods were applied to the identification of 5957 commercial, plant-based products sold in 37 countries and distributed in all six inhabited continents it was revealed that a substantial proportion (27%) of the products were adulterated. The highest levels of inauthentic samples were in products from Australia (79%) and South America (67%), and the lowest from Asia (23%). Some other levels in specific countries were Brazil (68%), India (31%), USA (29%), and China (19%) [166]. Note that this analysis probably doesn’t distinguish the substitution of a different, unapproved, plant part of an “authentic” plant, which would generate an “authentic” DNA analysis, and thus may be an underestimate of the level of criminal fraud. In a subsequent analysis, when 2386 plant-based products from 37 countries were analyzed chemically, 27% were adulterated in some manner, particularly samples from the United Kingdom (37%), Italy (31%), and the United States (27%) [167]. The absence of product consistency between batches of a specific product from the same manufacturer was also noted. These are further examples of the inability of the natural product manufacturing industry to self-regulate for the healthcare benefit of the patient and the equanimity of the practitioner [168–171]. A more concise example relates to the experimentally determined content of melatonin products in the United States marketplace.

Melatonin (*N*_b_-acetyl-5-methoxytryptamine) is produced nocturnally in the pineal gland in mammals [172] and is also available commercially as a dietary supplement to induce sleep. Although, from a clinical perspective, it is not approved by the United States Food and Drug Administration for any medicinal purpose. A recent study of melatonin-containing products in the United States revealed that 22 of 25 products were inappropriately formulated, some even including cannabidiol [163]. In a fascinating review, Khan and colleagues have recently provided a stunning, detailed summary of how “fraudsters” (their polite term!) carry out their numerous insidious methods to specifically *defeat* analytical systems aimed at quantifying the ingredients in dietary supplements [164]. Importantly, the authors indicate a range of widely marketed products most susceptible to adulteration for economic benefit, placing the patient in an impossible situation. Dietary supplements are a very significant industry in the United States, growing at 9.7% in 2021 to a retail value of $12.35 bn with direct sales increasing by 15.8% [173]. At the 10–27% level of incidence, that represents $1.235–$3.33 bn annually in fraud perpetrated on patients in the United States alone.

These on-going situations regarding the content of TM/CM products, locally within almost every country in the world, and on a wider basis with respect to internet acquisitions, reflect a low level of scientific expectation and intent for a finished, health beneficent product. That paradigm must change for both the patient and the practitioner. Sponsored by government and industry and working with the technical expertise of academia, prioritized TM/CM products must be evidence-based [174]. For the majority in the world, currently around 6.0 billion patients, little has changed with respect to their access to a reliable traditional medicine in the past 4000 years. For the rest of the world, patients are subject to the performance vagaries of complementary medicines and phytotherapeuticals (vide infra) [162–164]. This unacceptable breadth and depth of ongoing international fraud against patients makes the need to establish Q.S.E.C.A. as a baseline response indeed dire.

The World Health Organization has been urging a science-based approach to ensuring the quality of traditional medicines for over 20 years with the long-term goal of standardization and regulatory harmonization [174]. Paradigms must be shifted in this context to provide experimental evidence for quality, safety, and efficacy. The primary obligatory shift applies to the almost universal paradigm of “doing nothing”. Very few countries in the world, in either the Global North or the Global South have initiated structured research initiatives on their traditional medicines, phytotherapeuticals, or dietary supplements where the clear aims are product quality, safety, efficacy, and consistency for the primary benefit of the patient. The ongoing situation of *caveat emptor* continues to reflect an exceptionally dysfunctional marketplace for patients and practitioners in all international settings [47, 58, 62, 66, 75].

## A Linguistic paradigm in traditional medicine

The language used in traditional medicine must be accurate and strong. There is a paradigm involved with the continued and widespread use of two terms and their concepts which should be avoided when research on traditional and complementary medicinal plants is being discussed, namely “herbs/herbal” and “validate”. Herbs, plants that are used as condiments or in cooking, may indeed have medicinal properties, and garlic, capsaicin from chiles, and curcumin from turmeric, are well-established examples. However, the vast majority of the 25,791 plants used worldwide as traditional medicines in numerous systems are not used for culinary purposes. Consequently, the persistent use of the term “herbal medicine”, particularly by Western-trained physicians, is a blatant mischaracterization of the function and the predominant origin of traditional medicines (trees and shrubs) and therefore serves to maintain an irreverent, dismissive paradigm. That undermines the *status quo* and diminishes the value of traditional medicines as a vital healing modality in the numerous systems which either underpin, or are, the primary healthcare of most (> 74%) of the world’s population. Use of the term by scientists in the field therefore propagates the mythical paradigm that these plants have no medicinal value and is inhibitory to acquiring respect for meaningful funding to gather the evaluative scientific evidence to explore the biological and clinical effectiveness for inclusion in integrated healthcare programs for billions of patients. The preferred terms to use are therefore “traditional medicine” or “complementary medicine”, or “plant-based medicine”.

Closely related, and also originating in the philosophies and on-going myths of traditional medicine use, the term “validate” is frequently misapplied when discussing the assessment of a well-established, traditional medicinal plant preparation in an appropriate bioassay system as it implies prior experimentation. To be transparent, a long history of the traditional medicinal use(s) of a plant (or plant mixture) is exceptionally useful information, especially if it originates from multiple diverse systems, cultures, or regions, and has been recorded in some consistent manner over time. However, it is *not* evidence-based in contemporary science as WHO has proposed should occur. There is little or no knowledge as to the affirmed identity of the originating plant(s), or the reproducibility of the preparation method from the past records, or how those parameters relate to geographical differences in plant origin. Thus, defining accurately the plant and developing the reproducible preparation of an extract with a known (analyzed) chemical profile for therapeutically targeted biological evaluation represents a *new* experiment. Explicitly, it is *not* a “validation” of clinical efficacy for the historical use of the plant as there is no prior observation of a clinical or even biological response with that specific preparation [47, 62, 66]. Using the term “validate” therefore represents a “logical fallacy” in which the basic assumption, that an experiment is being repeated, is false.

The concept of eons-old perceived safety and efficacy is merely the first of more than a dozen “myths” of TM which must be dispelled. In themselves they evoke erroneous paradigms which represent a scientific and philosophical bias inhibiting the quality of the research questions being applied [58, 62]. To avoid that preconceived bias, explorative experiments based on a specific, characterized, reproducible TM/CM preparation, supported by chemical analysis, and with biological test systems of high relevance to the reputed traditional use, are preliminary “assessments” or “evaluations”. Importantly, as an integral aspect of the establishment of any biological activity or effectiveness, it is critical that the *same*, analytically well-defined, preparation is used for in vitro and in vivo pharmacological, pharmaceutical formulation, and toxicological experimentation, and eventually for clinical assessment. Characterization of the material that is *actually* being tested at the time of the biological or clinical test is an essential element for establishing reproducible biological data on a batch-to-batch basis [86].

## Enhancing patient product selection

Given the unacceptable purchasing options for patients, trust in the system which regulates and produces the manufactured product must be restored [175]. Patient centering is a prime focal element in the development of Industry 5.0 (5IR). That will necessitate a major paradigm shift in information and quality control systems to prioritize, protect, and assure the health care expectations of the patient and thereby renew faith in the marketed products. The applicable manufacturing and product regulations and the research substantiating an individual product must put the patient first and center, *not* the manufacturers.

Patients are now far more contextual and deliberate in their traditional medicine and phytotherapeutical investments. Thus, affirmed sustainability, fair trading acquisitions, Good Agriculture-Collection Practices, Good Laboratory Practices, Good Manufacturing Practices of their operations, and meeting international standards of performance are evolving in decision-making by the patient for product acquisition. For now, and for the future, how can these attributes of quality in processing be assured for the patient through traceability in a farm/forest to pharmacy supply chain? One approach is the use of blockchain technology which provides a digital ledger for recording the individual parameters at each process step. This information is then cryptographically secured and available to each participant in the chain, including, at the end, the patient [176–178]. The wine [179, 180] and agri-food [181, 182] industries are avidly exploring the pertinence and implementation of blockchain technology to track, in this immutable manner, the journey of an authentic product from farm/winery to the marketplace. Blockchain traceability processes for tea tree oil have recently been established in Australia [183].

The wide introduction of an immutable quality assessment of traceability for TM/CMs at each production and distribution stage from source (field or forest) to marketplace (store or on-line) using blockchain technologies [66, 184, 185] through a product QR code is essential. This would frame the market in favor of the patient, as both quality and content can (and must!) be established at each step in the journey. The practice would answer some pertinent (currently unanswerable) questions along the market chain. How old is that plant material? Where was it sourced? How? Where has it traveled, and where was it processed, packaged, and stored? What were the applied quality control parameters beyond macroscopic identification? What is its established shelf-life? Imagine if the *trust* for consistency in a traditional or complementary medicine was equal to that of your favorite bar of chocolate!! Why isn’t that “ideal” the consensus worldwide goal for government regulators and for the manufacturers? Those self-regulated companies which focus on the ethical presentation of quality natural products will benefit significantly economically and be trusted by the patient. It is well beyond a time for this transformational paradigm change across this manufacturing sector [8]. Systems to carry out this transition are beginning to be implemented [186].

There is an important consideration to be pondered. Namely, are natural products and traditional medicines “performing” for societal benefit as they should be? Or are they just “existing” within a conventional and well-framed paradigm? Have those resources regarding the plant and their historical use been optimized for patient benefit? Clearly, the answer is “No”. In that instance, there is a very basic ethical and sustainability need to establish and implement meaningful, reproducible, and monitored standards of practice and product performance for natural products to enable enhanced and assured patient benefit. This implies several important paradigm shifts, some of which are elaborated subsequently. They include a greater role in primary drug discovery through the establishment of regional compound libraries (accounting for intellectual property rights issues), the enhanced, traceable quality control of sustainable traditional and complementary medicine products. This should result, in some countries, in a new class of standardized, safe, and effective bioactive natural products which can be approved expeditiously and, importantly, economically, on a broad global basis. While not approved as a classical prescription drug, such sanctions for marketing would improve patient care in an affordable manner for many countries and would also develop an enhanced level of trust in the accessible products. Singly, this dramatic shift of prioritizing benefits for the patient would shatter the paradigms for many aspects of TM/CM development.

What are the health benefits and production criteria for those products? How will they be approved? Where, and how will those products be marketed? What medical claims will be allowed? Indeed, what are the implications in healthcare globally for a series of traditional or complementary medicines that have been demonstrated to be safe, effective, consistent, and sustainable? Will that begin to bridge the vast gap for better quality products for most patients? Will that enhance the possibility for more integrated healthcare programs? Will there be economic benefits for a healthier society? The challenges are truly substantial, yet necessary to be addressed, if modification of the present paradigms is to enhance healthcare directly. This is especially the situation as the synthetic medicines based on fossil fuels become no longer accessible and replacements need to be developed as a new aspect of bioprospecting.

An international initiative for contemporary, science-based regulations and their implementation for both traditional medicines and phytotherapeuticals is desperately needed. It is a core element of placing, as the highest priority, the fundamental expectations of the patient, that the plant-based medicine will be safe and that it will “work”. In addition, there is the need for the enhanced formal reporting, pharmacovigilance, of a demonstration of either toxicity, lack of effectiveness, or drug-drug interaction from a traditional medicine [187]. Patients, practitioners, regulators, and manufacturers must be clear with respect to a medicinal preparation that has both safety and efficacy and distinguishable from preparations where those attributes are challenged, or where there is unambiguous evidence to recommend that a particular plant be withdrawn from the marketplace.

The outcomes of TM/CM research must equate to living pharmacopeial standards and scientifically relevant regulations for content and biological outcome that are vigorously enforced through post-marketing surveillance. Analytical resources should be in place and deployed which monitor the products in the local marketplace, forcing sub-standard products to be removed with meaningful penalties enacted when there is evidence of criminal fraud. This paradigm shift divests the industry of control of the parameters for product standardization and places it in the arena of scientifically regulated products. It should provide a guaranteed health benefit for the patient and contribute to a restoration of trust in the TM/CM marketplace. It is recognized that such an evidence-based approach is both expensive and impractical for more than several hundred traditional medicines from various systems. However, since a healthy population reliant on the assured quality of traditional medicinal agents is critical in many emerging economies to develop initiatives for growth, investment in safe and effective medicinal agents based on sustainable sourcing is a highly desirable new paradigm [8, 48, 62, 91, 188]. Collaborative research programs between academic laboratories, industry, and regulators can develop reproducible, cost-effective methods for TM/CMs quality control processes which will meet the demands of Q.S.E.C.A. and enhance Medicines Security for the patient [48, 58, 59, 66, 91].

## Regulating the quality of traditional medicines

Establishing, and affirming through reproducibility, the appropriate regulatory standards for a TM/CM based on information systems, botany, chemistry, and biology is a complex and demanding process [91]. These analytical considerations are significant challenges to be overcome *en route* to an appropriate analytical standard protocol for a single plant or a more complex matrix. Widely distributed, biologically active metabolites (PAINS), such as catechols and quinones [189] and metabolites active in a very broad array of bioassays (IMPS), such as quercetin and gossypol [190], as well as rhodamine analogs, curcumins, and resveratrol [191] should not be considered as potential biomarker compounds for standardization. Those metabolites regarded as IMPS and PAINS mislead and/or conceal the determination of the true bioactive principles and consequently *cannot* serve as solo pharmacopeial or regulatory biomarkers for a particular medicinal plant. Consequently, dereplication systems for the dominant IMPS and PAINS must be included as an integral aspect of the functionality assessment of all TM/CM preparations [47, 66].

Clarity regarding the identification of the bioactive metabolites related to the traditional use of the plant must therefore take place, as well as an awareness of the potential for the presence of bioactive gene products from non-reproducible endophytic sources [192]. When a medicinal plant or other organism has more than one recommended use in traditional medicine, perhaps in different systems, discernment of the individual active principle(s), and their development as a biomarker, must be intimately associated with the specific intended use(s). A single, regulated bioactive standard cannot be used for the multiple proposed clinical uses of a medicinal plant preparation without a specific biological determination in a therapeutically relevant bioassay. Only then will the patient be receiving a standardized product that correlates with their expectation of a health benefit [108].

A holistic and integrated approach for TM/CM quality control is important for developing the initial standards for a safe and effective preparation. It should be based on integrated information systems, macro-, micro-, and DNA-based signature elements, coupled with metabolomics, and reproducible in vitro and in vivo biological profiling [91]. Rationalized and focused qualifying, individualized standards can then provide accessible parameters to develop contemporary, patient-centered, regulatory requirements and provide a simplified, robust, reproducible, and cost-effective industrial protocol to predict accurately the safety and effectiveness and thusly provide a consistent product for the patient. Post-market approval surveillance by an authorized agency is an essential element to establish and subsequently maintain patient trust in a product, supported throughout by blockchain technology. The analytical and spectroscopic techniques being employed to generate chemical fingerprints in traditional medicine have been reviewed and their importance in establishing standards discussed with examples [162].

An important, frequently overlooked, aspect of both regulatory control and sustainability is how to rationalize a complex traditional medicine mixture of plants which may possess a highly variable sustainability profile; summarily put, are all the constituent plant and other materials required for efficacy and consistency? Or can select plants be eliminated, substituted, or rationalized in terms of sustainable sourcing, while still respecting and providing the holistic recommendations of the traditional medicine system of origin? At that point, what are the parameters for the reapproval of a modified preparation.

From a biological functionality perspective, it is the assessment of these metabolites and the optimization of the accessibility of the most valuable which will increase in significance as the losses of biodiversity and the prevalence of fossil fuel energy issues coalesce. The molecules from natural sources that are basic for our nutritive health, our treatments, and our appearance, are produced through carefully controlled biosynthetic processes whose enzymatic pathways are being avidly explored, now at the gene level [109]. Concurrent efforts focus on understanding the regulation and expression of the processes and whether they can be modulated predictably. Genomic studies in bacteria, delightfully, revealed that the traditional approach of culturing an organism in one or two media does not reveal the full metabolite potential of an individual organism, typically, a bacterial genome possesses many uncharacterized biosynthetic gene clusters, potentially indicating new classes of metabolites to be assessed for biological potential [193]. These hitherto “silent” or “cryptic” pathways, which assuredly exist in plant genomics, although they may be harder to detect due to biosynthetic pathway fragmentation, will undoubtedly provide new chemical entities of biological significance. Optimizing the outcomes of these known and hidden metabolite pathways through heterologous transfer into other organisms (bacteria or plants) provides access to these new biosynthetic pathways [194]. The metabolomic result is then displayed through mass spectrometric analysis as interrelating webs of compounds showing both the major and minor components as a series of pathways that to a limited extent may also proffer aspects of biosynthetic interrelationships.

## Defossilization

According to the United Nations Secretary General, António Guterres, at the Davos Meeting in January 2023: “Today, fossil fuel producers and their enablers are still racing to expand production, knowing full well that their business model is inconsistent with human survival. This insanity belongs in science fiction, yet we know the ecosystem meltdown is a cold, hard scientific fact.” He added: “We must act together…..to stop our self-defeating war on nature.” This is an accurate assessment at several levels for many areas of “business” which produce or utilize fossil fuel-derived chemicals as their core starting material or which are integral to their final product. The paradigm shift away from fossil fuels in the energy and other sectors is termed “defossilization” [195].

Production of fossil fuels and the synthetic products derived therefrom, including fine chemicals and prescription and OTC medicinal agents, will continue until access to either oil or coal resources “runs out”, i.e., becomes too costly to extract and derive a profit from. According to a 2019 report by the Millennium Alliance for Humanity and the Biosphere, for oil shale and oil sands that situation could occur by 2052 and for coal by 2090 [196]. Environmental controls to limit overall sea level and climate change will therefore also deeply affect access to non-renewable, oil- and coal-derived chemicals and solvents. The conclusion is that strategies, including a more robust circular bioeconomy and more extensive carbon capture technologies, will be necessary to meet the distinct need to “defossilize” energy requirements, with the associated profound challenges regarding the production of synthetic and natural drugs [197].

Although progress in the applications of green chemistry have been impressive in the past 30 years [198], and now include some considerations of life cycle assessment [199], it is certain that there will be a significant impact on the ability to synthesize certain approved medicinal agents in support of existing health care systems. This will necessitate that, over time, 10–20 years maximum, a major paradigm shift to chemical feedstocks and reagents which are recyclable or derived from green carbon sourcing (e.g., renewable biocatalysts and isolated enzymes). These opportunities will engage synthetic organic chemistry to create new chemoenzymatic pathways to key chemicals avoiding the use of non-reusable chiral reagents, and where possible utilizing whole vegetables or other plant or microbial resources (no enzyme isolation needed!) to conduct, in high yield, both chiral and achiral reactions [200].

Chemical libraries of functionally diverse, stable, reproducible, and recyclable enzymes with high levels of substrate promiscuity that can be readily integrated into eco-friendly organic reaction sequences for known and new chemical entities, will be an important long-term contribution of studies of the enzymes of natural product biosynthesis as feedstocks decline. Time is of the essence and accelerating the discovery and development of new medicines for global and local diseases, continuing to develop environmentally friendly, readily biodegradable plastics, cosmetics, fertilizers, herbicides, and insecticides, and evaluating new foods and crops with enhanced nutritional value to feed a starving world should now be the core prospecting activities for organic and natural product chemists. Exogenous chemicals introduced into the environment must be appropriately functional, and demonstrated to be nondepleting, nontoxic, and nonpersistent [67, 199]. That will require extensive commitments to collaborate with various biologists and ecologists to examine environmental impact prior to marketing approval.

The successful introduction of “green” chemistry philosophies and practices [67] is only a start, not an endpoint. It is an abiding process that will involve continuous expansion, refinement, and creative development as affordable access to fossil fuel products recedes. Defossilization studies will also require a quite different approach regarding the selection of drugs that are amenable to greener processing demands, thereby minimizing and eventually eliminating a reliance on fossil fuels, while attempting to maintain access [201]. This scenario creates paradigm-shifting challenges for evolving biowaste research initiatives and the associated economic developments, likely resulting in a restructuring of major pharmaceutical entities to assure chemical feedstocks.

Profound investment in developing alternative sourcing for key precursor compounds for organic synthesis will be needed in the next 20 or so years to establish a fundamental base for the million-ton synthesis of starting materials and to assess which bulk and specialized chemical reagents will have a sustainable future [202]. Many specialized chemicals will undoubtedly fall by the wayside in this period, and thus reagent accessibility will likely become a factor limiting AI/ML assessed synthetic strategies in industry and academia. Detailed long-term analyses will be necessary to generate a target list of highly desirable chemical entities and new synthetic approaches designed for their availability, for which AI/ML will be a critically strategic adjunct. Biopharmaceuticals will require special attention, and indeed pharmaceutical companies, in collaboration with biowaste processing specialists, have a powerful opportunity to lead in these research efforts through a combination of chemical and biological approaches. Garnering timely investment requires a demonstrated market outlet, and the development of a production infrastructure for defossilized medicinal agents. As mentioned, like the fine chemicals, some of these synthetic and semisynthetic agents will be “lost” due to an inability to produce them in a sufficiently “green”, economical, and sustainable manner. That will create gaps to be filled by natural products through opportunities for niche replacement from a sustainable source.

Biocircular economy strategies would also benefit from feasible industrial side-product and waste streams and could help enhance biorefinery development and reduce supply-chain anomalies [203, 204]. It should also be a highly productive research endeavor to create the access to fundamental building blocks which, based on ML, can be utilized for the synthesis of either needed existing or new compounds [205]. A recent example focused attention on the selective degradation of lignin, the dominant source of the 3′,4′-oxygenated-C_6_C_2_-phenylethyl scaffold on the planet, for the purpose of fine chemical development [206–208] and new product synthesis [209, 210]. These goals were achieved through the formation of an acetal derivative of 4-hydroxy-3-methoxyphenylacetaldehyde [211, 212] which could be modified to form quinazolinones, tetrahydroisoquinolines (e.g., tetrahydropapaveroline), and *N*-phenethylindole derivatives providing new “hit” molecules [205].

Based on the continuous evolution of circular economies for selected bioresources, for example in potentiating the microbial products and amino acids of biowaste for organic chemical use, natural products sourced from those degradative processes will evolve as potential replacement starting materials for a new phalanx of approved medicinal agents. Balancing the emerging technologies of the Fourth Industrial Revolution, the 4IR, with the added facets of sustainability, resilience, and a personal focus from the 5IR with respect to all medicinal agents, but particularly those of natural origin, will be the core activity for maintaining the security for medicines and pursuing enhanced new products for the patient. Clearly, medicinal chemistry and synthetic drug development and manufacture will need to be fundamentally restructured based on access to starting materials that are derived through recycling and purposing, the “green” processing of biowaste and carbon capture, not those from a fossil fuel. The enormity of this transition cannot be overstated.

Research centers of science and technology excellence are necessary to embrace the critical environmental, agricultural, medicinal, and nutritional issues whose evolution and associated research demands increasingly impact our daily lives. Only diverse, well-funded, creative, and collaborative expertise will have the capacity to solve these significant realities and concerns for the future sourcing of medicinal agents. Eco-awareness initiatives will involve creative synthetic and natural products chemistry programs, while integrating cultural knowledge as more aggressive efforts evolve for medicinal agents, for biodegradable plastics, for biofuels, for multiuse enzymes, and for enhanced standards for the production and content control of foods and TMs to protect and treat the customer/patient.

As indicated earlier, the staggering volume, occurrence, and deleterious effects of biologically broad, human and veterinary prescription medicines on release as unwanted waste through various pathways into the environment has been well-summarized [213–215]. The incidence and metabolism of over-the-counter products in the environment has received less attention [216]. Therefore, more intensive ecological and environmental assessments of medicinal agents will be needed as an integral component of the drug approval process, including their metabolic degradation in the soil, in sewage treatment, and in selected aqueous environments. In addition, the impact of these chemicals and their metabolites on the fauna which consume the water from these contaminated environments requires more detailed evaluation [217, 218]. Exploration is needed on the metabolic impact of the absorption of these chemicals, particularly regarding the phytoremediation required from exposure to organometallics [219], into the metabolic profiles of medicinal plants and compounds incorporated through proximity to the rivers and streams which are carrying these pharmaceuticals. Also to be considered are those chemicals (natural and synthetic) distributed onto soils in fertilizers and insecticides for newly cultivated medicinal plants, and what effect that may have on their metabolic profiles [220, 221]. A distinct advantage of natural products is that over billions of years their metabolism and impact in a specific local environment, and their effects on flora and fauna have achieved a balance, in which biodegradation has a long, integrated evolutionary history. What occurs when new synthetic compounds are brought into an established environment necessitates investigation as a life cycle assessment, as mentioned earlier.

If natural products based on historical or newly discovered biological or medical use are indeed to make a more significant, quality-controlled, and resource and biologically optimized impact in society as an improved and approved adjunct within integrated healthcare systems, there are important questions of sustainability to be addressed [85]. In addition to changes in metabolite profile [32], they include does changing the environment of a plant have the possibility to cause deleterious effects in the new location? Numerous examples already exist where this has occurred through the introduction of various invasive species into non-historical locations, and the economic and environmental damage that causes [222]. In addition, as the production of natural product-based medicinal agents increases over time, it is important to ask whether they indeed do have a lower carbon footprint than synthetic drugs? Is there a tipping point where the assessed environmental impact of a traditional medicine preparation is greater than that of a synthetic drug? It is another aspect of the “performance” outcomes of ecopharmacognosy presented earlier which merits evaluation and evolution of assessment parameters.

Illicit natural drugs must also be considered with respect to both the damage and waste in the environment that their processing causes. For the first time, in 2022, the United Nations World Drug Report began to examine the environmental impacts of the illicit production of synthetic and natural drugs [223]. Although relatively minor on a global basis, locally in the producing areas, the impact can be highly significant based on the illicit growing and processing of cocaine, opium, and cannabis. The direct impacts include energy consumption, soil and water pollution from the heavy use of pesticides and insecticides for plant growth, the solvents and other chemicals necessary for processing the natural products. Also to be considered are the impacts of discarding the residual chemicals into rivers and streams without treatment, and of the deforestation which ensues to create the necessary secluded space for cultivation, and air pollution. It should be noted, however, that deforestation for coca bush cultivation is less in acreage than the cleared land created for new food crop and grazing range development in the Western Amazon [223]. The typically remote processing sites for illicit drugs indicate and assure the absence of any regulated oversight for waste disposal. In-field drug plant eradication programs also have deleterious outcomes since ongoing illicit production results in further deforestation and loss of the rich biodiversity and an increase in fragile and fragmented ecosystems. As a measure of the environmental effects, since 2001 about 741 million acres of primary forest have been cleared for coca cultivation alone [224]. That staggering volume corresponds to about 40% of the size of the continental United States or Australia. It is recognized that the carbon footprint of indoor cannabis cultivation is 16 to100-fold that of in-field cultivation and is increased through enhanced CO_2_ availability [223]. For cocaine production, the carbon footprint is estimated to be 2,600 times that of sugarcane, with the global emissions for in-field processing comparable to that of almost 2 million gasoline-powered cars (~ 8.9 million tons of carbon) per year.

## Illustrations of L.O.V.E.

The optimization of known and effective resources to potentiate yields, reduce toxicity, simplify, and ameliorate the impact of processing, and access reusable compounds are some of the many ways that will require implementation to assure and increase the effectiveness of each biological material. Thus, for the future, L.O.V.E., optimizing what Earth has to offer humanity, is not an option. It is an obligatory commitment to all future generations for their succeeding generations. Earlier optimization examples include enhancing product yields, “greening” metabolite processing, and the repurposing of established medicinal agents for new biological indications [225]. More recent examples come from the field of natural cancer chemotherapeutics and from the application of gene modulation.

The precursor for the semisynthesis of the anticancer agent etoposide is (-)-deoxypodophyllotoxin (DP) which is derived from the roots of the Himalayan mayapple, *Podophyllum hexandrum* Royle (Berberidaceae). A more sustainable, higher-yielding source was sought. Coniferyl alcohol (CA) is an intermediate towards DP, and the genes for the biosynthesis of CA from L-phenylalanine, together with those for the conversion of CA to DP, were engineered into the fast-growing *Nicotiana benthamiana* Domin (Solanaceae) and the large leaves processed to afford 0.71 mg/g dry weight of DP [226]. In this approach, issues regarding the availability from a slow-growing source and the level of production from that non-sustainable source are addressed. As a second example, the biosynthesis of the potent anti-multidrug resistant antibiotic platensimycin from *Streptomyces platensis* SB12026 was enhanced through examining the regulatory genes [227]. Specifically, deletion of the pathway-specific transcriptional repressor gene *ptmR1* led to a fermentation yield of 0.75 g/L [228]. In the case of the Hsp90-inhibiting antibiotic macbecin, originally characterized from *Nocardia* sp. No. C-14919 [229], specific mutations abrogated the genes encoding for enzymes introducing those functional groups identified as responsible for installing toxicity into the scaffold and led to a product which was 80-fold more active than the parent metabolite [230].

Optimization in terms of metabolite deployment for expanding patient benefit includes the repurposing (also called repositioning or re-tasking) of known, approved medicinal agents [231, 232]. An essential caveat is that the resource is sustainable. Artificial intelligence and machine learning (AI/ML) are now playing an increasingly important and decisive role in compound identification and biological target selection [233–236]. In the past, alkaloids such as colchicine [237], pilocarpine [238], capsaicin [239], scopolamine [240], and quinine [241], among others, have each found new clinical uses. More recently, the need for new antivirals based on the COVID-19 epidemic [242] has been highlighted, as well as initiatives for neurodegenerative diseases [243], and cancer [244]. These searches have been aided also by diverse introductions of AI/ML, including for the enhanced biological activity of traditional Chinese medicines [245]. Repurposing is a burgeoning area wherein data-based metabolite libraries can be evaluated rapidly against new binding sites, and comparisons made with known active compounds, thereby reducing waste and resources resulting more highly focused in vitro screening. It is another vivid demonstration of bioprospecting optimizing the already identified valuables of Earth.

Conscious, mindful development of optimization, as both a philosophy and a practice, dovetails with the myriad aspects of a circular economy, which, in part, focuses on the fully functional use of all sourced materials, including through the strategies of biotechnology and the minimization of “waste” [246]. For example, the leaves of *Agave sisalana* Perrine ex Engelm. (Asparagaceae) are processed to form the hard fiber sisal, but that represents only 3–5% of the total weight of the plant. The waste contains steroid saponins with potentially many diverse uses in foods, cosmetics, and pharmaceuticals, and thus efforts have been made to enhance their isolation, resulting in high levels of saponin recovery as an aspect of biocircular economic development [247].

Repurposing transcends many aspects of optimizing uses of energy and materials. Plastics are an enormous global issue from many perspectives [248] and there are widespread global efforts underway to recycle various types of plastic in the construction [249] and furniture [250] industries to reduce the reliance on native wood resources. As a further example of the repurposing of waste materials, in late 2023 the first transatlantic (London to New York) commercial flight took place with the use of a sustainable, waste-derived biofuel, and commitments for a vast increase in the use of this biofuel have been made [251]. Other biofuels under development may be corn-based [252]. However, whether there is adequate land and accessible water supplies to grow the volume of corn needed for an extended period remains a pertinent question [253].

## The impacts of technology

The contemporary paradigms and myths applied to traditional medicines must be regarded as transitional. There is a presumptive myth that when paradigms are “shifted”, a new, better paradigm is inherently created [254]. That may be the intention, but that is not always the eventual outcome. The currently applied analytical and biological tools (in silico and in vitro), and the available datasets on natural products and their physical and spectroscopic properties, have transformed the depth to which an extract can be assessed, and the rapidity and eco-friendliness of that process [255]. As a result, the canards of perceived analytical constraints of assessing complex plant mixtures have largely been abrogated [256], replaced by almost unfettered analytical capacity. Indeed, there is now a plethora of information to be analyzed, particularly through liquid chromatography/mass spectrometry and nuclear magnetic resonance techniques. A critical human activity, following AI data analysis, has become an assessment of the appropriateness and accuracy of the spectral data interpretation provided by AI [257]. Can a molecule suggested by AI be definitively identified as a natural product? Or are there other considerations? Who is considered responsible for the data interpretation? [258]. Recalling the Zen tale of the rider and the fast-moving horse [259], it cannot be “the horse”, control of decision-making must befall the intervention of “the rider” [8]. Succinctly, the outcomes from an AI-based analysis must engage human experience and expert knowledge in the data verification process.

The renowned Chicago architect Louis H. Sullivan is noted for the euphemism “Form ever follows function, and this is the law.” First discussed in relation to his insightful observations of nature in the eclectic *Lippincott’s Magazine* in 1896 [260], the phrase is perfectly pertinent regarding the contemporary societal relevance of natural products. In the context of the continuous integration of technological innovation into natural product practices, and in developing the research programs which will be most meaningful for the patient and the greater society, it is essential that the functions for the future of natural product research be well-defined, as financial resources are limited. Only then can the associated actions to establish a framework for prioritization be proposed and developed. What do we want the foci in this 4IR and 5IR epoch to be under the auspices of the Quintuple Helix, and while simultaneously addressing Sustainability Development Goals, with the human experience as a central, prioritized consideration which reflects human progress for the future? What are the respective priorities and are they different based on societal needs at the variable global levels of economic and healthcare system development? Are there overarching global issues to be addressed?

Natural product scientists, with their diversity of scientific backgrounds, each have enhanced, fundamental responsibilities for natural products taxonomy, chemistry, biology, and ethnopharmacology, data acquisition and analysis to conduct and promote research that will benefit and sustain humanity for the generations to come. The relevance of the available technologies in diverse collaborative research teams will differ. Many refinements will be made in the research goals and practices as the local and global environment changes over time. Deep work and creative thinking will be required to focus on the needs for a sustainable and healthy human experience for our descendants by continuously challenging the extant practices and paradigms. Put another way, if your ongoing research in philosophical style and technological involvement has not changed in the past five years something is probably wrong. Einstein considered that creativity was seeing what everyone else was seeing and then thinking what no-one else had thought. That is precisely where natural product scientists stand. The requisite collective information is becoming globally available at an increasing rate and is most likely within your handheld device. In other words, everyone has access to all the needed background and data sets. Parity of information access clarifies that it is human creativity and the subsequent actions that will differentiate the long-term outcomes of innovation and economic development in various societies.

Historically, education systems are responsible for providing access to various learning experiences. For the future, a paradigm shift, beginning in kindergarten, will be required to accentuate the primary functions of educational institutions to enhance levels of creativity and critical thinking with respect to the essentials of the Sustainable Development Goals in promoting sustainability in society in an ethical manner. Within the collaborative opportunities of the Triple Helix, it will also involve the continuous and evolving aspects of balancing the implementation of innovative technologies and the wisdom of their extended impact and compassion for the environment and future generations.

## Cyberecoethnopharmacolomics and the fourth and fifth industrial revolutions

In 2019, the author introduced the term “cyberecoethnopharmacolomics” to place emphasis on the need to recognize that beyond “ecopharmacognosy”, the sustainable philosophies and practices applied to pharmacognosy, was a wealth of interwoven and interdependent areas of science, technology, society, and the environment, that needed to be embraced in an integrated discussion to bring real societal value and benefit to research on biologically active natural products research through a concerted, strategic process [47, 62, 66]. A prior discussion from 1987 had pointed out that, with a veritable plethora of available technologies integrated into natural product research advancements, “Pharmacognosy……is *the* most high-tech pharmaceutical science” [107]. That statement is even more accurate today, as it represents the full integration and application of all the philosophies, practices, and technologies available at any one time to enhance what the patient eventually experiences for their prophylaxis or healing, what environmental damage needs to be ameliorated, what research materials need to be recycled, and what organisms need to be sustained and regenerated. For what purposes are these productivity-oriented technologies of the 4th Industrial Revolution (4IR) being applied with respect to optimizing benefits for the patient and for the planet? The initiatives of the 5IR, relating to co-robotics, sustainability, becoming more human centered, socially relevant, and having various aspects of resilience together respond to defining the role of natural products in a technologically integrated milieu.

Industry 4.0 (4IR) is focused on the development of a set of technologies and how they are deployed with respect cyber-physical systems and industrial supply chains with a view to enhancing production efficiency, i.e., value creation for industry [261–263]. Three facets were glaringly missing, the handling of industrial waste and environmental protection, sustainability, and the “human” element of co-performance by the workers [263, 264]. The Fifth Industrial Revolution (Industry 5.0) seeks to remedy that situation through a focus on the synergistic, creative interactions of humans and machines [265, 266]. One aspect of this is the development of a perceptive and autonomous workforce, the cobots, operating through collaborative and cooperative processes, not competition [267]. Based on an extensive AI background, environmental pollution and biological waste should be reduced, particularly as clarity regarding the potential further applications of such waste becomes apparent [267]. “Digital twins” will become more important, where all the aspects of a human process towards a product or activity are recognized digitally by an intelligent, robotic device with additional considerations to minimize environmental pollutants. Such developments are standard for automobile manufacture and have been employed in the rapid operation and analysis of hundreds of organic syntheses in narrow timeframes [268]. Another focus of Industry 5.0 is, similar to the additions of the Quadruple and Quintuple aspects to the Triple Helix of cooperative and collaborative activities, intercepts with the human aspects regarding societal relevance and sustainability [269]. Succinctly, while Industry 4.0 has industrial enhancement as its primary theme, Industry 5.0 has human creativity, sustainability, and resilience (vide infra) as dominant driving elements.

Several concepts of the 4IR have a direct impact on natural products discovery and development, and on the strategies for enhancing the quality of traditional medicines [66]. Beyond the vast impact of biotechnology, three technologies merit particular emphasis, namely, big data, additive manufacturing, and robotics. The open availability and utilization of “big data” sets are inherently broad and global. For involvement in natural products research, a significant challenge is data acquisition from the myriad of silos from all over the world. The Global Natural Products Social Molecular Networking portal provides fundamental information accrual regarding the mass spectral analysis of metabolites through a crowdsourced curation approach and living curation of data to improve accuracy of recommended identification [270]. The outcome, metabolite networking, can propose structures for known metabolites in a matrix without isolation through the successful access to variously dispersed databases, linking spectral patterns with compound classes and eventually clusters of closely related individual metabolites [271].

Extending the rapid access to fundamental information systems correlating taxonomy, chemical structures, spectral data, and biological activities in silico and in vitro is more challenging and yet, for future natural product development activities, is essential. Preferably this would occur through a single, universally accessible portal [272]; a deep-dive, Google-like system for all that is known about natural products. This opportunity, in consort with government agencies and relevant innovative industrial groups, would transform, reshape, and define new priorities and strategies to disclose and characterize medicinal and biological agents from nature based on new approaches. Through machine learning algorithms, it will identify potential compounds for in vitro biological assessment following in silico binding analysis, for repurposing established compounds for hitherto unconsidered activities (vide supra), or for site-specific synthetic adaptation to enhance a bioactivity based on interpretations of network pharmacology, as well as the clinical uses for specific disease biomarkers derived from metabolomics. Hand-held and drone-manipulated chemical sensors are having a pronounced impact in agriculture and in chemical and illicit drug production site identification [273, 274]. Those uses must be expanded in application to monitor medicinal plant distribution through species-specific unmanned aerial vehicle-borne sensors [275].

Systems such as the Global Biodiversity Information Facility under development at the Royal Botanic Gardens in Kew, England provide access to extensive acquired data on all types of life on Earth. Yet, with respect to plants, they only represent 21% of the vast herbarium and botanic garden records from about 53,000 datasets in 1600 institutions, access being limited in some countries by CBD/Nagoya Protocol considerations [45]. The evolving data acquisition and analysis systems at Kew will facilitate where alternative plant resources might be located, and eventually where the biosynthetic gene clusters for specific metabolites are to be found, including those previously undisclosed.

Accumulating data is merely a starting point, integrating, evaluating, and purposing for a societal niche benefit are the essential outcomes as fundamental aspects of the optimization Earth’s resources. For example, data banks for genomic information on microbial systems [276], and specifically on microbial biosynthetic gene clusters [193, 277–279] are an essential aspect of mining biosynthetic pathways for new metabolites and improving the production of metabolites from a single genome, e.g., through regulatory gene deletion [228], as discussed earlier.

Industry 4.0 (4IR) also emphasizes the potential developments of additive manufacturing. With the advent of bench-top equipment, the days of prefabricated laboratory glassware are probably numbered as custom-designed, laboratory-made equipment for multi-step organic synthetic processes [280], for targeted natural products isolation, and for in-field bioassays, based on microfluidic processing [281] becomes mainstream. The creation of microfluidic, in-field bioassay systems is very important for the assessment of traditional medicines either in the forest or under cultivation. These manufacturing techniques also extend to the development of personalized delivery systems for biologically active compounds and sustainable metabolites [282–284].

Silica-based glass is not biodegradable and thus has a significant environmental footprint external to the development of a circular economy. However, a biomolecular, peptide-derived glass was recently described which is more eco-friendly [285] and is based on the chemical modification of peptides to enhance their thermostability [286]. The products were transparent, biocompatible, biodegradable (in vitro and in vivo), biorecyclable, and could be 3D-molded.

The vast area of automated devices and robotics already encompasses chromatographic and analytical spectral instruments in natural product isolation and characterization, systems for the programmed synthesis and characterization of target molecules through AI/ML-assisted retrosynthetic analysis, as well as for biological assessment [287]. Integrated systems of functional robots are operating in pharmaceutical laboratories making recommendations for human consideration and performing a variety of activities [288, 289]. They, the cobots, will soon take their place in academic laboratories performing, among other tasks, plant and microbial culture processing and sample preparation functions.

For future developments applied to natural products there are probably five principal outcomes. The first relates to a deeper commitment to dedicated programs that focus directly on benefits to society coupled with a prime consideration of sustainability. One might see this in a discovery program where ML applies parameters, including sustainability and background knowledge, regarding a particular series of plant or microbial materials based on prior data to develop a rational action plan for the analysis, acquisition, and assessment of materials of potential interest. Secondly, it is anticipated that a higher level of cooperativity and synergy would develop as more applied, functionally capable, robots are introduced into laboratories. Thirdly, the human element of creativity and innovation will be continuously assessed by ML leading to a better understanding of the processes and pitfalls and enhanced decision-making. The fourth aspect for development will be the expansion of data systems that can be integrated into process decisions, and this aspect will likely be enhanced as aspects of Open Science and broader access occurs with respect to original and processed data [290]. Automated recommendations for the structure determination of synthetic reaction products and metabolites [291] will evolve rapidly following more efficient separation systems and product analysis, as well as proposing synthetic pathways [292]. In addition, there is the opportunity through AI/ML to bring significant focus to the large-scale assessment of compound matrices, in silico, against biologically important disease-related enzymes [235, 293]. Remembering that these uses are centered towards human and societal issues. Finally, the term “resilience” is particularly applicable through Industry 5.0 and relates to ongoing data integrity and retaining authenticity through significantly enhanced data security parameters, both locally and in large datasets. It is well-recognized that data security, for example, in academia, is either poor or non-existent, particularly if scientists are working outside their laboratory/office environment. Eventually, resilience will also apply as the parameters of blockchain technology are applied to the immutable quality of the medicinal and biological agents in the retail market.
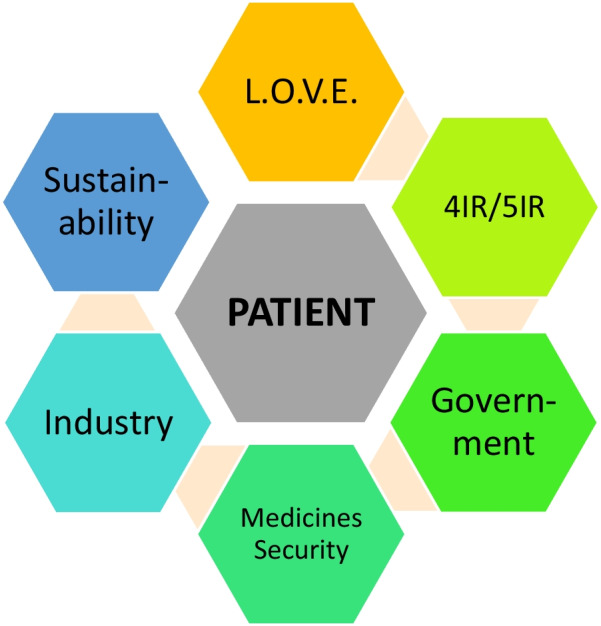


## The contemporary paradigms are failing—action is needed

In earlier iterations of aspects of this discussion [8, 85], and in this presentation, many existing natural product and traditional medicine paradigms have been indicated, and they are frequently limiting the ability to optimize the resources of Earth for future generations. Patients, practitioners, and natural product scientists are asking the pertinent question “….which way ought we to go from here?” [59] for optimum patient and environmental benefit. Due to the continuing recalcitrance and insouciance at both government and industrial levels, a worldly choice remains: i) accept the *status quo* with all its foibles for the patient, or, ii) encouraged by Robert Frost’s eponymous poem, take the road less travelled [294], and cut a new pathway through the thicket of inaction to a patient-oriented place in line with Industry 5.0. As the management consultant Edwards Deming declared in *Out of the Crisis*, “Quality begins with intent”, adding “We have lived in a world of defective products. It is time to adopt a new philosophy” [295]. At the global level, it has been made clear that traditional and complementary medicines are “defective products” when they cannot be trusted by the patient (or the practitioner) for their quality, safety, efficacy, consistency, and accessibility. Thus, for the patient, that long overdue time for decisive paradigm change in assuring the quality of TM/CM products for patient benefit has arrived.

Evolutionary philosophical and practical changes to improve TM/CMs for the patient through collaborations between academic research groups, industry, practitioners, government health agencies, and regulators are essential as a high priority to improve healthcare outcomes. A negotiated way forward to change the paradigms for patients globally must be found. As the architect and philosopher R. Buckminster Fuller has indicated, “To change something, build a new model that makes the existing model obsolete.” Coupled with the comments of Deming, the new model, derived from a series of initiatives and higher-level targeted research directions, locally and globally, must prioritize quality in natural products for the care of the patient as an aspect of healing the planet in a sustainable manner, and thereby optimizing our natural resources for the medicinal and biological benefits of the global society.

From the preliminary discussions outlined in this article and in several earlier publications, presented herein are forty initiatives to transform the contemporary paradigms to a model for patient-centered care which has the philosophical strength, the core ethical and scientific values, and which actively promotes the evidence-based, patient-centered, sustainable development of TMs/CMs as an international standard. To design and expand this new model for the development of natural products and ethnopharmacology, Government agencies, industry, researchers, and practitioners must promote and implement:

1. Medicines Security as a unifying aspect for Global Health
2. putting the benefit for the patient first in the ethical and scientific considerations of TM/CM development
3. garnering financial support for a ten-year, structured, global program for TM/CM research and standardized product development
4. local, regional, and international collaboration between governments, academia, and industry to enhance the quality control of medicinal plant products
5. the harmonization of TM/CM regulations within and between regions for individual and complex plant preparations
6. “living” pharmacopeial standardization relevant to safety and bioactivity for the patient and practitioner
7. a class of approved, standardized, safe, and effective natural medicinal agents
8. the standardization of TMs/CMs based on biomarkers relevant to the healthcare intent
9. patent-free, traditional medicine-based products
10. the inclusion of standardized, safe, effective, and sustainable TM/CM products in the essential medicines lists of countries
11. enhanced, immutable quality control practices based on patient-centred, pharmacopeial and regulatory standards
12. the traceability of TM/CM products from farm/forest acquisition to marketplace using blockchain technology, emphasizing financial, quality, and sustainability parameters
13. the unambiguous and complete labeling of TM/CM products
14. the acquisition of country specific and worldwide data on the use of plants and animal products in traditional and complementary medicine
15. programs to establish the sustainable sourcing of the prioritized plant materials utilized in TM/CMs
16. sustainability parameter determinations for the 400 most important medicinal plants, including the mapping and monitoring of medicinal plant resources, through field and remote determinations, coupled with the impacts of environmental change
17. the acquisition, collection, and analysis of volume usage data for TMs/CMs within a country or region
18. sustainable practices for plant collection, utilization, and laboratory experimentation
19. scientific studies which rationalize complex TMs to maintain sustainability and patient accessibility
20. products meeting international standards for the acquisition, manufacture, storage, and marketing of TMs to establish patient trust
21. studies to determine the stability and shelf-life of specifically formulated products
22. post-market surveillance of products and the reporting of substandard “cluster” outbreaks
23. product labeling with a “sustainability quotient”
24. the evaluation of accessibility in a particular cultural environment to assure continuing patient access
25. the standardization and registration of internet TM/CM products in the originating country
26. an international network of information sources compiling the adverse and synergistic effects of TMs/CMs and prescription and OTC medications, together with their contraindications
27. the elimination of ineffective TMs from continued marketing as a patient/practitioner protection
28. the collection of genetic data, the development of seed banks, conservation gardens, cultivation initiatives for the most important medicinal plants in select locations
29. the equitable, negotiated acquisition, consolidation, fundamental analysis, and global accessibility of traditional knowledge, and the associated botanical, chemical, and biological information on natural products for AI/ML analysis and research development.
30. strategies to address unmet local and worldwide disease needs based on sustainable natural resources
31. studies of the effects of environmental change on the metabolite profile of the most important TMs/CMs
32. plant tissue regenerative studies for threatened and valuable medicinal plants
33. the development of regional centers of excellence to establish TM/CM content control parameters
34. the sharing of technologies employed in TM/CM research through publications and on-site training programs
35. the integration of contemporary technologies based on the 4IR, the 5IR, and the concepts of the Quintuple Helix into TM/CM research and quality control
36. the regeneration of “waste” lands to produce important medicinal plants
37. a dramatically enhanced role for the existing and new WHO Collaborating Centers for Traditional Medicine for the education of TM/CM researchers, manufacturers, regulatory personnel, and practitioners
38. awareness education programs for all involved stakeholders aimed at eliminating the myths of TM
39. inclusion of TM/CM philosophies, practices, and scientific developments in professional academic curricula for physicians, dentists, pharmacists, and nurses
40. advanced TM/CM degree programs focused on botanical, chemical, biological, clinical, data-, and gene-based research programs.

## Conclusions

Each day, the news is replete with reports of unexpected floods, massive fires, warmer seas and oceans, and disappearing lakes and rivers bearing witness that these are indeed the burgeoning environmental outcomes of the Anthropogenic Era. The dramatic and rapidly evolving experiences for societies all over the world in relation to their natural resources (food, essential oils, medicines, cosmetics, fragrances, biocides, gums, waxes, and other commercial natural materials) will continue, and may accelerate as we reach and then exceed the 1.5 °C average temperature increase for which the world is destined very soon. These increasingly intense interactions between environmental change and humanity must be confronted as a present, deepening, on-going reality which now cannot ever be avoided. The projections beyond 2050 for the transformational flooding of many islands, coastal areas, and major population centers around the world are dire. Coupled with other climate modulations, these vast, incomprehensible, and unimaginable changes will have a myriad of unpredictable lifestyle outcomes, in the spectrum and prevalence of local vector-borne diseases, including neglected tropical diseases, and in the availability and metabolite consistency of the natural resources required for numerous diverse clinical and societal purposes. The comment of the United Nations Secretary General cited earlier clarifies that “human survival” depends on an urgent response to the ongoing crisis.

These changes inherently provide vibrant new areas for creativity and intellectual opportunity in natural products chemistry and biology at the interface of Industry 4.0 and 5.0, as pertinent questions are posed by society for a livable future. Priorities for continuous action will shift over time as meaningful responses are made and situations ameliorated or hopefully at least managed. Ecopharmacognosy was proposed in 2012 as a strategy of philosophy and practice to unite the concepts of sustainability and green chemistry with natural products chemistry and biology. Cyberecoethnopharmacolomics emerged to reflect the high levels of integrated science, technology, and the omics required contemporaneously to address significant natural product-focused issues and needs in society and operate within the combined parameters of the 4th and 5th Industrial Revolutions. Post COP28, as transitioning from fossil fuels engages, pursuing all the integrated technologies, intensely, for the optimization of Earth’s natural treasures must also enhance the ongoing efforts to establish Medicines Security as a necessary baseline for quality assured, safe, efficacious, and available TM/CMs and synthetic and biological medicinal agents for the benefit of all patients, while embracing the sustainable elements of the associated ecopharmacognosy practices and philosophies.

As natural product scientists, our primary role is now to address those societal needs for the optimization of natural products on a continuing basis. We do this through planning and action, developing new questions, and then creating and innovating new strategies and applied solutions to optimize the valuables of Earth. That is our primary teaching on creativity for the future. We must do this for all those descendants who will follow. *For the future of humanity, failure in this endeavor is not an option*. There cannot *ever* be a Plan(et) B for humanity and the other creatures of our deeply interconnected Earth. We are already headed, rapidly in terms of timeframe, towards a more profound tipping-point, the experience of the disaster of a gutted Earth, in an evidently non-reversible manner. “Transitioning away” from fossil fuels is only a negotiated pathway step. It is not a long-term, paradigm-shifting solution. It represents enormous organic chemical challenges and opportunities for the provision of natural and synthetic medicines that are fundamental to global health care, and without timelines and performance parameters it may not succeed. Only by responding more urgently and positively with extensive investment to engage the evolving priorities, can we maintain a sustainable environment for future generations, even if a steady-state or declining world population can be realized.

As these ventures into an unknown world down an untrodden pathway evolve, it is vital that the successes within Medicines Security are effectively transmitted to other scientists worldwide, and to a waiting and increasingly concerned society. It is a profoundly exciting era for organic chemistry and all the natural product sciences to “step-up” and assume an appropriate role in healthcare for all societies. Our creative and practical skills in applying the existing and new technologies to develop and implement innovative strategies for the optimal utilization of the Earth’s resources can make a significant difference for the future of humanity, locally and globally. Collaboratively, we *must* find effective strategies to optimize the sustainable natural resources for all patients and consumers of a diversity of products.

The forty areas of paradigm change in traditional medicine for “promotion” outlined here necessitate for their implementation a manifestly enhanced level of commitment to urgent action from international agencies, governments, manufacturers, and natural product scientists. Outmoded paradigms must be dropped. Myths must be debunked. Unexplored directions, creating new pathways must be taken. We must be the pioneers for creative, sustainable natural resource applications. New local, regional, and international alliances involving governments, industry, and academia must be built. Profound, 180° shifts in philosophy and strategy are necessary in major corporations to place the Earth and its people before profit, or any profit will inevitably be lost to compensate for the induced environmental damage. Enhancing the health and welfare of societies must be the central goal and be made sustainable and regenerative. A country cannot thrive with an unhealthy populace in an unhealthy environment. The extensive and deep environmental changes that are rapidly unfolding require active leadership and action. A vision for Medicines Security can only occur through gatherings of donor nations to sponsor specific initiatives formulated by the World Health Organization, the United Nations Industrial Development Organization, and other pertinent international agencies, government health ministries, industries, and global philanthropic foundations. Initiatives must focus on patient-centered health care based on sustainable, natural medicines and strategies for defossilization. The expertise is already in place in many developed and emerging countries to begin to effect these inquisitive transformations. To promote and coordinate these activities, a Global Association for Medicines Security is urgently needed which can bring focus, highlight the central issues, and develop a strategic research and financial plan for programs to address the fundamental needs to shatter the existing paradigms, with input from all the relevant national and international stakeholders. The function is to establish systems for the long-term benefit of the patient and the planet beyond 2050. This singular global commitment can ensure Medicines Security for all, as originally embraced by the World Health Organization Constitution. After all, this is not for us, it is for our descendants, and always will be.

## Data Availability

There are no experimental data associated with this article.
